# Synovial Fluid‐derived *Micrococcus Luteus* G18 Exacerbates Osteoarthritis Progression by Promoting Chondrocyte Degradation via TLR2/JNK/AP‐1 Signaling Pathway

**DOI:** 10.1002/advs.202514220

**Published:** 2025-11-16

**Authors:** Tingtao Chen, Qingwei Zeng, Tangchang Xu, Xinyue Qi, Kaiyi Li, Jing Wei, Liang Hao

**Affiliations:** ^1^ Department of Orthopedics The Second Affiliated Hospital Jiangxi Medical College Nanchang University Nanchang Jiangxi 330006 China; ^2^ Jiangxi Province Key Laboratory of Bioengineering Drugs School of Pharmacy Jiangxi Medical College Nanchang University Nanchang Jiangxi 330031 China; ^3^ Institute of Orthopedics of Jiangxi Province Nanchang Jiangxi 330006 China; ^4^ Institute of Minimally Invasive Orthopedics Nanchang University Nanchang Jiangxi 330006 China; ^5^ Jiangxi Provincial Key Laboratory of Spine and Spinal Cord Disease Nanchang Jiangxi 330006 China; ^6^ National Engineering Research Center for Bioengineering Drugs and the Technologies, Institute of Translational Medicine, Jiangxi Medical College Nanchang University Nanchang 330031 China; ^7^ School of Life Sciences Nanchang University Nanchang Jiangxi 330031 China; ^8^ College of Food Science & Technology Nanchang University Nanchang Jiangxi 330031 China

**Keywords:** articular cavity microenvironment, microbiota, *Micrococcus luteus*, osteoarthritis, TLR2/JNK/AP‐1 signaling pathway

## Abstract

Traditionally considered a sterile environment, the articular cavity has this perception overturned with advances in multi‐omics technologies—microbial communities are increasingly identified in once‐thought sterile tissues, making the articular cavity a potential microbial niche. However, the presence and role of intra‐articular microbes in osteoarthritis (OA) remain rarely studied. Synovial fluid microbiomes of OA patients at different stages are analyzed via 16S rRNA high‐throughput sequencing, and the results show that microbial diversity is positively correlated with OA progression, with the microbiomes dominated by Proteobacteria, Firmicutes, and Actinobacteria. Culturing yields 145 strains, with *Micrococcus luteus (M. luteus)* significantly enriched in advanced OA. In OA rats, intra‐articular transplantation of synovial microbes or *M. luteus* G18 exacerbates cartilage damage. Mechanistically, *M. luteus* G18 activates the TLR2/JNK/AP‐1 pathway via surface peptidoglycan, disrupting chondrocyte homeostasis to inhibit extracellular matrix (ECM) synthesis and promote degradation. These findings not only provide the first comprehensive evidence of the joint cavity microbiota but also unveil *M. luteus* G18 as a microbial driver of OA progression. This study reshapes the understanding of OA pathogenesis and opens new avenues for microbial‐based diagnostics and therapeutics—pointing toward a previously overlooked dimension of joint biology that deserves further exploration.

## Graphical abstract

Traditionally considered sterile, the articular cavity is now recognized to harbor microbial communities influencing osteoarthritis (OA). Integrating 16S rRNA sequencing and culturomics, *Micrococcus luteus* is enriched in advanced OA. Notably, *M. luteus* G18 exacerbates cartilage degeneration via TLR2/JNK/AP‐1‐mediated ECM disruption, revealing intra‐articular microbiota as previously unrecognized pivotal contributors to OA pathogenesis with promising diagnostic and therapeutic potential.

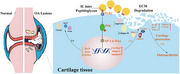

## 1 Introduction

Osteoarthritis (OA) is a widespread chronic degenerative disease of the joints, primarily marked by the degradation of cartilage, the formation of osteophytes and synovial inflammation.^[^ ^1^, ^2^ ^]^ As a major contributor to chronic pain, mobility restriction, and physical disability, OA currently affects more than 595 million individuals worldwide, posing a considerable challenge to global public health.^[^ ^3^, ^4^ ^]^ The pathogenesis of OA is multifactorial, involving genetic predisposition, aging, sex, obesity, and mechanical stress.^[^ ^2^, ^5^ ^]^ However, current models fail to fully elucidate its complex mechanisms. Identifying novel molecular and cellular pathways is therefore essential to overcome existing therapeutic challenges and to uncover new diagnostic markers and treatment targets.

OA pathogenesis is intricately linked to inflammation, metabolism dysregulation, imbalance between extracellular matrix (ECM) synthesis and degradation, and aberrant immune responses.^[^ ^6^ ^]^ Critically, dysregulation of the joint microenvironment is increasingly recognized as a key pathological mechanism underlying OA progression.^[^ ^7^, ^8^ ^]^ The joint microenvironment constitutes a highly dynamic system comprising synovial cells, chondrocytes, synovial fluid, immune cells, and ECM components, which interact to maintain homeostasis and joint health under normal conditions. However, disruption of joint microenvironment homeostasis due to infection, inflammation, or other stimuli initiate and perpetuate OA progression, with inflammation playing an indispensable role.^[^ ^9^, ^10^ ^]^ A hallmark of microenvironment imbalance involves the elevated expression of pro‐inflammatory cytokines, including tumor necrosis factor‐α (TNF‐α), interleukin‐1β (IL‐1β), and IL‐6, while anabolic factors like SRY‐box transcription factor 9 (Sox9) and aggrecan (Acan) are downregulated. This process instigates a sequence of reactions within the mitogen‐activated protein kinase (MAPK) signalling pathway, resulting in the activation of matrix metalloproteinases (MMPs) and the promotion of cartilage degradation and subchondral bone remodeling abnormalities.^[^ ^11^, ^12^ ^]^ Notably, *Zhang* et al. demonstrated that elevated TNF‐α levels in the joint microenvironment activate the c‐Jun N‐terminal kinase (JNK) signaling pathway, which further induces the expression of inflammatory mediators IL‐1β, TNF‐α, and MMP13, accelerating ECM degradation and osteophyte formation, ultimately exacerbating OA progression.^[^ ^13^ ^]^ Thus, these findings highlight a pathological feedback loop wherein inflammatory mediators disrupt the joint microenvironment, degrade the cartilage matrix, and impair subchondral bone metabolism—collectively driving OA progression.

Traditionally, the joint cavity was considered a sterile microenvironment. However, advancements in high‐throughput sequencing and omics technologies have challenged this view and progressively identified microorganisms within the joint cavity. Specially, *Qiu* et al. collected shoulder cartilage samples from 23 patients undergoing joint replacement surgery and found that 74% of the samples contained *Acinetobacter* species and *Oxalobacteraceae* family members, as identified by 16S rRNA sequencing, indicating a low‐abundance but consistent microbial presence in the shoulder joint tissue.^[^ ^14^ ^]^ What is more, *Cheng* et al. integrated 16S rRNA sequencing, electron microscopy, and culturomics to identify diverse microbial populations in synovial fluid from patients with advanced rheumatoid arthritis, revealing the presence of viable bacteria and suggesting that joint microbial dysbiosis may disrupt chondrocyte glycosaminoglycan metabolism, thereby promoting cartilage degradation and accelerating arthritis progression.^[^ ^15^ ^]^ Nonetheless, research on joint cavity microbiota remains scarce, and the precise composition, functional roles, and pathological impact of these microbes in OA are still largely undefined—highlighting an urgent need for deeper investigation.

In this study, we comprehensively analyzed synovial fluid from 40 patients with OA using high‐throughput sequencing to profile the microbial composition and crucially identified *Micrococcus luteus* G18 (*M. luteus* G18) as a potential key pathogen through culturomics. Furthermore, by employing MIA‐induced OA rat models and in vitro chondrocyte assays, we conclusively demonstrated that *M. luteus* G18 drives cartilage degradation and accelerates OA progression via activation of the TLR2/JNK/AP‐1 signaling pathway. Collectively, our findings present compelling mechanistic proof for the pivotal role of joint‐resident microbiota in OA pathogenesis and importantly offer a transformative perspective for the development of microbiota‐based diagnostic biomarkers and targeted therapeutics in clinical practice.

## 2 Results

### 2.1 Composition of Synovial Microbiota Shows Positive Association with OA Severity

**Figure 1:**
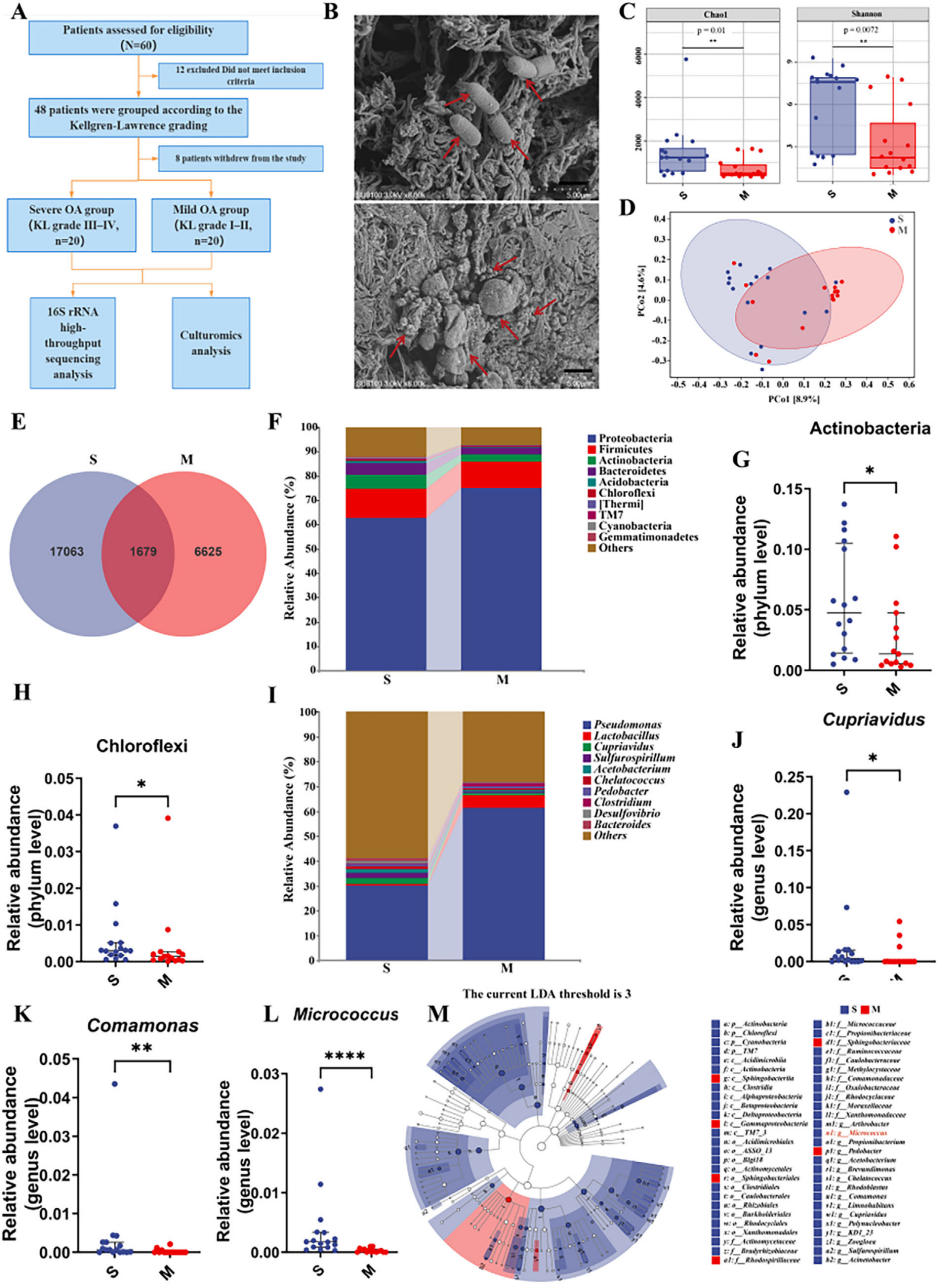
Recruitment of OA patients and synovial fluid microbiota analysis. A) Flowchart of patient enrollment and randomization. B) Scanning electron microscopy of synovial fluid (red arrows indicate bacteria); scale bar, 5.0 µm; magnification, 8000×. C) Alpha diversity (Chao1 and Shannon indices). D) Beta diversity analysis. E) Venn diagram of shared and unique taxa. F) Relative abundance of microbial phyla. G,H) Phylum‐level abundance of Actinobacteria and Chloroflexi. I) Genus‐level microbial composition. J–L) Genus‐level abundance of *Cupriavidus*, *Comamonas*, and *Micrococcus*. M) LEfSe analysis showing taxa significantly enriched (LDA score > 3) based on 16S rRNA sequencing. S: Severe knee OA group (n = 16); M: Mild knee OA group (n = 15). Data are presented as median (IQR); Mann–Whitney U test was used for statistical comparisons. *p < 0.05, **p < 0.01, ***p < 0.001.

To investigate the presence of microbial communities in synovial fluid and their potential correlation with OA progression, we enrolled 40 patients diagnosed with OA. Participant classification was conducted according to the K‐L grading criteria. Individuals with K‐L grades III‐IV were assigned to the severe osteoarthritis group (S group), whereas those with K‐L grades I‐II were categorized into the mild osteoarthritis group (M group) (**Figure** **1**A). No statistically significant differences were observed between the M and S groups in baseline demographic and clinical characteristics, including age, sex distribution, involved joint sites, and body mass index (BMI). However, the S group exhibited a markedly longer duration of disease progression (p = 0.003), accompanied by significantly increased radiographic severity compared to the mild group (*p <* 0.001; Table S1, Supporting Information).

Visualization of microbial components in synovial fluid was achieved through scanning electron microscope (SEM). The ultrastructural observations revealed a substantial presence of coccoid bacterial forms, along with a moderate population of rod‐shaped bacteria (Figure 1B), providing preliminary evidence of microbial colonization within the joint cavity. Subsequently, 16S rRNA gene sequencing was conducted for comprehensive taxonomic profiling of the synovial fluid microbiota. Analysis of alpha diversity demonstrated significantly higher values for Chao1 richness, Observed Species count, Shannon index, Simpson index, and Pielou's evenness in the S group compared to the M group (*p <* 0.05; Figure 1C; Figure S1A, Supporting Information), reflecting greater microbial richness and evenness associated with advanced disease stages. Beta diversity analysis revealed a clear separation in microbial community composition between the M and S osteoarthritis groups, as illustrated by ordination plots (Figure 1D; Figure S1B, Supporting Information), indicating distinct overall microbial profiles associated with disease severity. Venn diagram analysis showed a substantially higher number of unique OTUs in the S group than in the M group (17,063 versus 6625; Figure 1E). Analysis at the phylum level identified Proteobacteria, Firmicutes, Actinobacteria, and Bacteroidetes as the dominant bacterial taxa present across all synovial fluid samples (Figure 1F). It is worthy of note that there was a significant elevations in the relative abundances of Actinobacteria, Chloroflexi, Cyanobacteria and TM7 in the S group (*p <* 0.05; Figure 1G–H; Figure S1C,D, Supporting Information). At the genus level, marked compositional differences were observed between groups (Figure 1I). The genus Pseudomonas was more prevalent in the M group (Figure 1I; Figure S1E, Supporting Information), whereas Cupriavidus, Comamonas, and Micrococcus were significantly more abundant in the S group (*p <* 0.05; Figure 1J–L), with Micrococcus showing the most pronounced enrichment (*p <* 0.01). Moreover, the relative abundances of several pathogenic genera, including *Acinetobacter*, *Brevundimonas* and Prevotella etc. were elevated in the S group compared to the M group (Figure S1F–M, Supporting Information). Linear discriminant analysis (LDA) further identified multiple genera with increased abundance in the S group, including Acinetobacter, Prevotella, Micrococcus, Cupriavidus, Comamonas, Brevundimonas, and Propionibacterium (Figure 1M; Figure S1N, Supporting Information), suggesting a higher microbial burden and potential pathogenicity in patients with severe OA.

**Figure 2:**
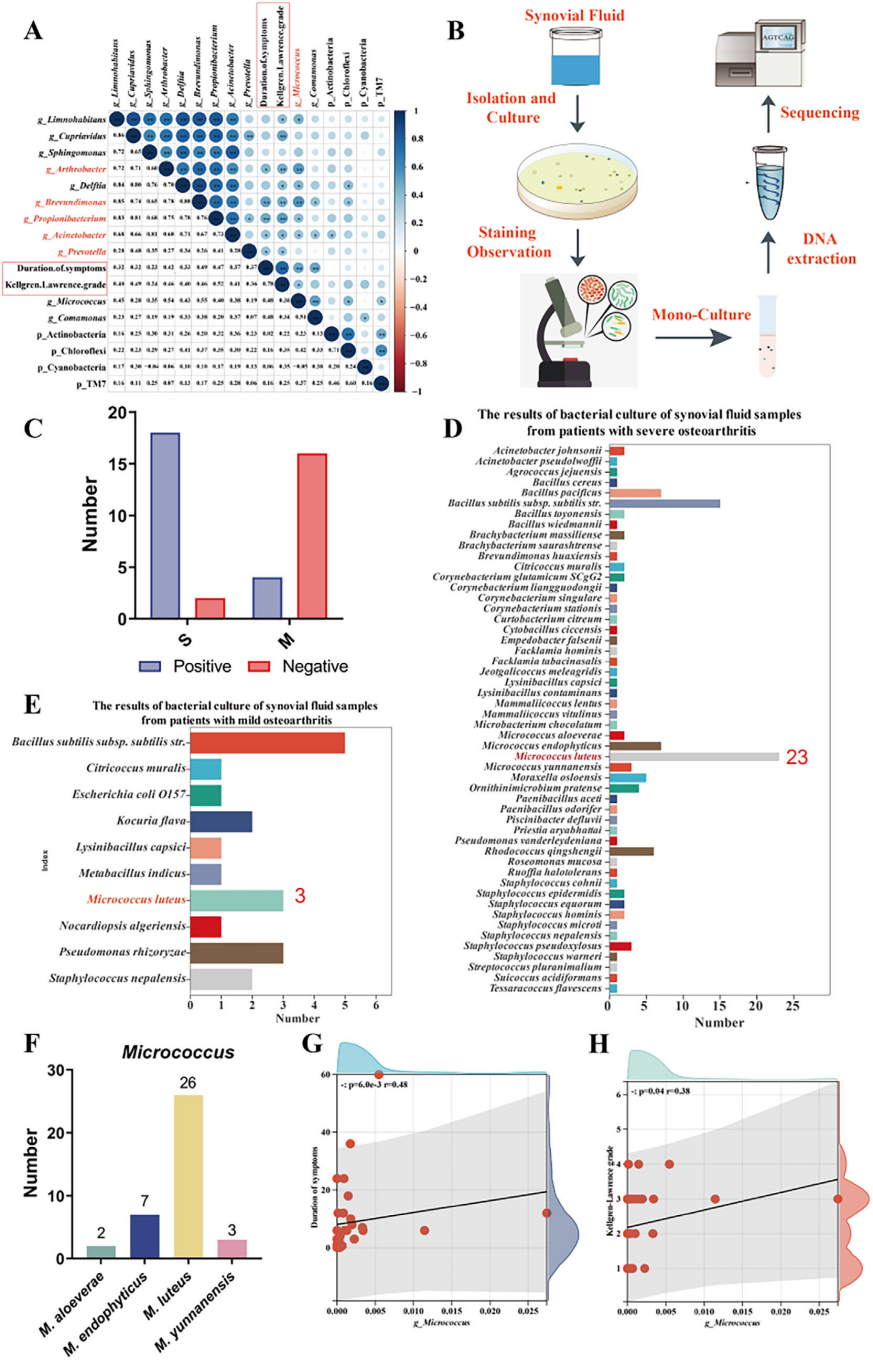
Culturomics analysis of synovial fluid in OA patients. A) Heatmap of correlations between clinical parameters and sequencing data (blue: positive, red: negative). (B) Culturomics workflow for synovial fluid. C) Summary of overall culture results. D,E) Culturomic profiles in severe and mild OA patients. F) Genus‐level abundance of *Micrococcus*. G,H) Linear correlations between *Micrococcus* abundance and disease duration or severity (S: n = 16, M: n = 15). S: Severe knee OA; M: Mild knee OA.

Spearman correlation analysis was conducted to explore the relationship between synovial fluid microbial profiles and osteoarthritis progression. Findings revealed significant positive correlations between the relative abundances of several microbial genera within synovial fluid and both the severity and duration of OA. Specifically, significant positive correlations (*p <* 0.05) were observed for the genera *Prevotella*, *Acinetobacter*, *Arthrobacter*, *Micrococcus*, *Brevundimonas*, and *Propionibacterium* (**Figure** **2**A). These findings suggest that an increased presence of potentially pathogenic bacteria may be closely linked to the onset and progression of OA. To further characterize the microbial composition in synovial fluid, a culture‐based microbiological analysis was conducted on 40 synovial fluid samples (Figure 2B). Among these, 55% (22/40) yielded positive cultures, with a markedly higher positivity rate in the S group (18 cases) compared to the M group (4 cases) (Figure 2C; Table S2, Supporting Information). In total, 145 bacterial isolates were cultured, representing 57 distinct species. Notably, 125 isolates were obtained from the S group, whereas only 20 isolates were recovered from the M group (Figures 2D,E; Table S3, Supporting Information). Among these, *Micrococcus luteus* and *Bacillus subtilis* subsp. *subtilis str*. were identified as the most abundant strains (Table S3, Supporting Information). Preliminary culture analyses revealed that the bacterial load in human synovial fluid generally ranged from ≈10^2^ to 10⁴ CFU mL^−1^, although considerable inter‐sample variability was observed. Integrative analysis combining high‐throughput sequencing, correlation analysis, and culture‐based data revealed that the genus *Micrococcus* consistently showed a positive association with disease progression across multiple analytical dimensions. Of particular interest, *M. luteus* emerged as the most frequently isolated strain within the *Micrococcus* genus, accounting for 26 isolates (Figure 2F), with a detection rate of 30% across all synovial fluid samples. Furthermore, *Micrococcus* abundance was significantly correlated with both clinical disease grading and disease duration (*p <* 0.05; Figure 2G,H). Based on these findings, we hypothesize that *M. luteus* may represent a potential core microbial species contributing to the progression of OA.

Collectively, these findings demonstrate a significant association between synovial fluid microbiota and OA progression. Notably, the high prevalence and strong positive correlation of *M. luteus* with disease severity and duration suggest its potential involvement in OA pathogenesis.

### 2.2 Transplantation of Synovial Fluid Microbiota from Patients with Severe OA Aggravates Joint Degeneration in a Rat Model

**Figure 3:**
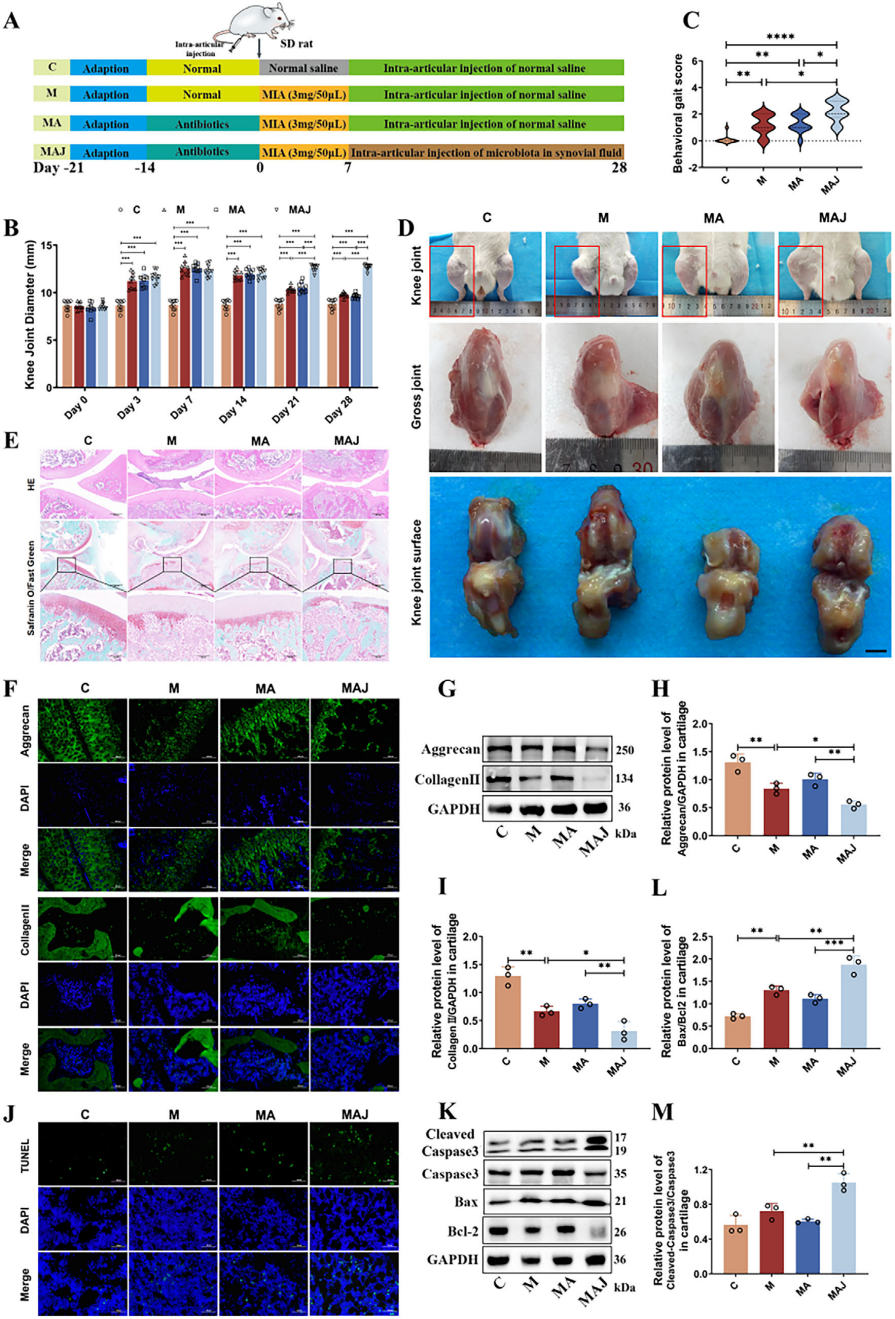
Synovial fluid microbiota accelerates knee OA progression in rats. A) Experimental design. B) Knee joint diameter measurements (n = 10 rats per group). C) Gait scores (n = 10 rats per group). D) Gross morphology and histopathology of knee joints. E) Histological analysis with H&E and Safranin O–Fast Green staining. F) Immunofluorescence of Aggrecan and Collagen II in cartilage. G–I) Western blot analysis of Aggrecan and Collagen II expression. J) TUNEL staining for apoptotic cells. K–M) Western blot of Cleaved Caspase‐3, Caspase‐3, Bax, and Bcl‐2 in articular cartilage. C: Control; M: OA model; MA: Antibiotic‐treated; MAJ: Antibiotic + OA synovial fluid microbiota (n = 3 per group). Data are presented as mean ± SD; statistical analysis by one‐way ANOVA. *p < 0.05, **p < 0.01, ***p < 0.001.

Clinical evidence reveals an association between synovial fluid microbiota composition and OA pathogenesis. To further elucidate the influence of synovial microbes on the progression of OA, we established an OA rat model via intra‐articular injection of MIA^[^ ^16^ ^]^ (**Figures** **3**A, S2A, Supporting Information). Subsequently, microbial intervention experiments were conducted using synovial fluid obtained from patients with severe osteoarthritis. Prior to OA induction, rats in both the MA and MAJ groups underwent a 2‐week pretreatment with a mixture of broad‐spectrum antibiotics to deplete endogenous joint microbiota.^[^ ^17^, ^18^ ^]^ This approach aimed to minimize interference from resident microbiota and to establish a controlled intra‐articular environment for evaluating the pathogenic or modulatory effects of exogenous microbial communities on OA progression. At the study endpoint, macroscopic examination revealed that C group rats maintained normal joint morphology, characterized by smooth joint skin, elastic periarticular tissues, uniform joint spacing, and unrestricted movement without friction or stiffness. In contrast, M group rats displayed joint swelling, reduced range of motion, joint rigidity, periarticular hardening, and narrowed joint space. Pain‐avoidance behaviors were also observed (*p <* 0.01; Figure 3B), confirming successful OA induction. Importantly, rats in the MA group showed joint pathology comparable to the M group, indicating that depletion of endogenous joint microbiota alone had minimal impact on OA progression. However, rats in the MAJ group exhibited significantly exacerbated joint swelling, osteophyte formation, further joint space narrowing, and markedly impaired mobility compared to the M group (*p <* 0.01; Figure 3B). Given the clinical relevance of pain in OA, we conducted gait analysis to evaluate nociceptive outcomes. The MAJ group exhibited a marked decline in gait performance relative to the M group (*p <* 0.05; Figure 3C), reflecting enhanced nociceptive responses and functional impairment associated with microbial exposure. Anatomical assessments supported these findings. Knees from the C group appeared structurally intact, with healthy, vascularized periarticular tissues and smooth, pale cartilage of uniform thickness. In contrast, knees from the M group demonstrated periarticular congestion, joint edema, roughened articular surfaces with focal discoloration and hyperemia, and classic OA features—including reduced cartilage transparency, surface irregularities, and focal erosions extending to subchondral bone. These pathological features were more pronounced in the MAJ group, including intensified hyperemia, joint swelling with poorly defined contours, severe cartilage discoloration, extensive defects, and disrupted cartilage continuity (Figure 3D).

Immunofluorescence analysis revealed that key structural proteins in articular cartilage, including Collagen II and Aggrecan, were unevenly distributed and markedly reduced in the M group (Figure 3F). Notably, the MAJ group exhibited more severe cartilage damage compared to the M group, characterized by aggravated ECM loss, further disruption of cartilage integrity and continuity, and a more pronounced decrease in Collagen II and Aggrecan expression (Figure 3E,F). These observations were corroborated by Western blot analysis, which demonstrated significantly reduced levels of Collagen II and Aggrecan in the M group (*p <* 0.01, Figure 3G–I), with an even greater reduction observed in the MAJ group. In addition, TUNEL staining and apoptosis pathway assays further confirmed exacerbated cartilage damage in the MAJ group. Specifically, TUNEL staining revealed a statistically significant increase in apoptotic chondrocytes within the MAJ group in comparison with the M group. A notable upregulation of Cleaved Caspase‐3 and the pro‐apoptotic marker Bax was detected, accompanied by a significant reduction in the expression of the anti‐apoptotic protein Bcl‐2, indicating enhanced apoptotic signaling activity (*p <* 0.01, Figure 3J–M). Collectively, these findings indicate that synovial fluid microbiota intervention exacerbated chondrocyte apoptosis and cartilage matrix degradation. Given the established role of inflammation in OA‐related pain and tissue damage,^[^ ^19^ ^]^ we next assessed serum concentrations of inflammatory cytokines and cartilage degradation markers using ELISA. Compared with the C group, the M group exhibited significantly increased concentrations of pro‐inflammatory cytokines IL‐1β, IL‐6, TNF‐α, as well as elevated levels of matrix metalloproteinase‐13 (MMP13) and the collagen type II degradation marker CTX‐II (*p <* 0.01). Importantly, the levels of these biomarkers were significantly higher in the MAJ group compared to those observed in the M group (*p <* 0.05; Figure S2B–F, Supporting Information).

In summary, intra‐articular administration of synovial fluid microbiota from patients with severe OA significantly aggravated joint inflammation, cartilage destruction, and pain in a rat OA model, thereby accelerating disease progression. These findings provide experimental support for a pathogenic role of synovial microbiota in OA and underscore their potential as therapeutic targets.

### 2.3 Synovial Fluid Derived from Severe OA Patients Increases Intra‐Articular Microbial Diversity in Rats

**Figure 4:**
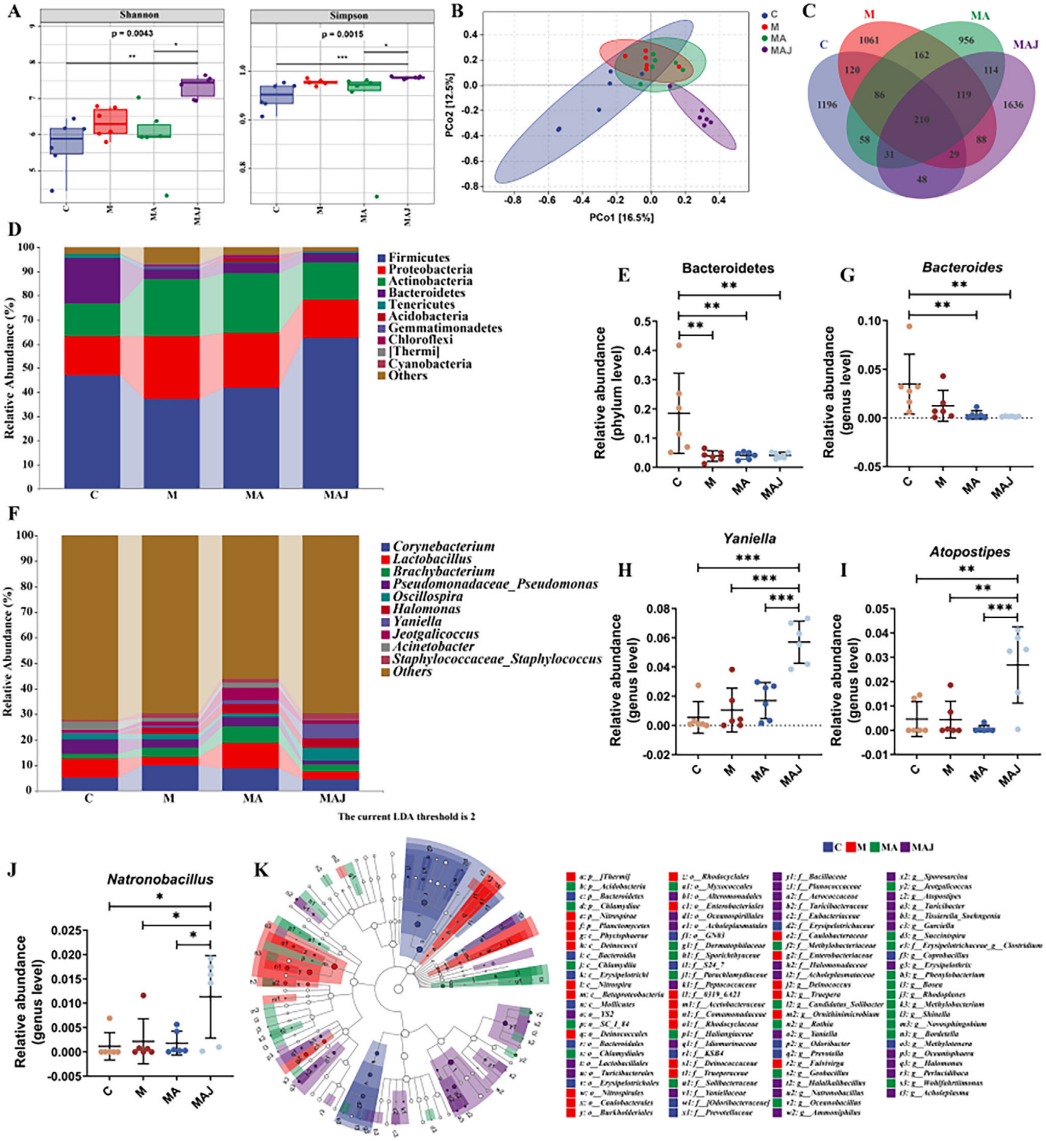
Microbial community analysis of the knee joint synovial cavity in OA rats. A) Alpha diversity indices. B) Beta diversity analysis. C) Venn diagram of shared and unique taxa among groups. D) Microbial composition at the phylum level. E) Phylum‐level abundance of Bacteroidetes. F) Genus‐level microbial distribution. G–J) Genus‐level abundance of *Bacteroides, Yaniella, Atopostipes*, and *Natronobacillus*. K) LEfSe cladogram illustrating taxa with relative abundance (circle size proportional to abundance). C: Control; M: OA model; MA: Antibiotic‐treated; MAJ: Antibiotic + OA synovial fluid (n = 6 per group). Data are presented as mean ± SD; statistical analysis by one‐way ANOVA. *p < 0.05, **p < 0.01, ***p < 0.001.

Previous results have demonstrated that synovial fluid microbiota from patients with S group exacerbates OA progression in rats. To further elucidate the underlying mechanisms, high‐throughput sequencing was utilized to systematically characterize the impact of microbial intervention on the diversity and composition of intra‐articular microbial communities in the rat knee joint. Our results revealed that the rat joint cavity harbors a rich and diverse microbiota. Compared with C group rats, the M group exhibited significantly increased α‐diversity indices—Shannon, Simpson, and Pielou's evenness indices—indicating a more complex microbial ecosystem in the joint cavity (*p <* 0.01; **Figures** **4**A, S2G, Supporting Information). Notably, alpha diversity was further elevated in the MAJ group following microbial intervention (*p <* 0.05; Figure 4A, Figure S2G, Supporting Information). Beta diversity analysis using both Principal Coordinate Analysis (PCoA) and Non‐metric Multidimensional Scaling (NMDS) revealed that the microbial community structure in the MAJ group was distinct from that of other groups (Figure 4B, Figure S2H, Supporting Information). Venn diagram analysis further demonstrated considerable differences in species composition across groups. Specifically, the observed count of unique OTUs was 1196 in the C group, 1061 in the M group, 956 in the MA group, and notably 1636 in the MAJ group, indicating markedly increased microbial diversity in the MAJ group (Figure 4C).

At the phylum level, the predominant taxa in the rat joint cavity included Firmicutes, Proteobacteria, Actinobacteria, and Bacteroidetes (Figure 4D). The relative abundance of Firmicutes was significantly increased in the MAJ group (*p <* 0.05; Figure S2I, Supporting Information), while Bacteroidetes was more abundant in the C group than in the other groups (*p <* 0.1; Figure 4E). At the genus level, the dominant microbial genera in the rat synovial cavity included Corynebacterium, Lactobacillus, Brachybacterium, Pseudomonas, Oscillospira, Halomonas, and Yaniella (Figure 4F). Compared with other groups, the MAJ group exhibited significantly increased abundances of several potentially pathogenic genera, including Bacteroides, Yaniella, Atopostipes, Natronobacillus, Halomonas, Salinicoccus, Staphylococcus and Streptococcus (Figure 4G–J, Figure S2J–M, Supporting Information). These findings were further supported by LDA, which confirmed significant differences in microbial composition among groups and highlighted specific taxa enriched in the MAJ group (Figure 4K, Figure S2N, Supporting Information).

This study is the first to comprehensively characterize a highly diverse microbial community within the rat knee joint cavity, resembling the complexity of gut microbiota. More importantly, OA progression was associated with significant alterations in joint microbial composition, particularly with increased abundance of pathogenic genera. The microbial intervention using synovial fluid from severe OA patients markedly enhanced microbial diversity and pathogen prevalence in the MAJ group. The results indicate that remodeling of the intra‐articular microbiota may represent a key mechanism underlying the accelerated OA progression observed in the MAJ group.

### 2.4 *M. luteus* G18 Isolated from Synovial Fluid Aggravates OA Progression in Rats

**Figure 5:**
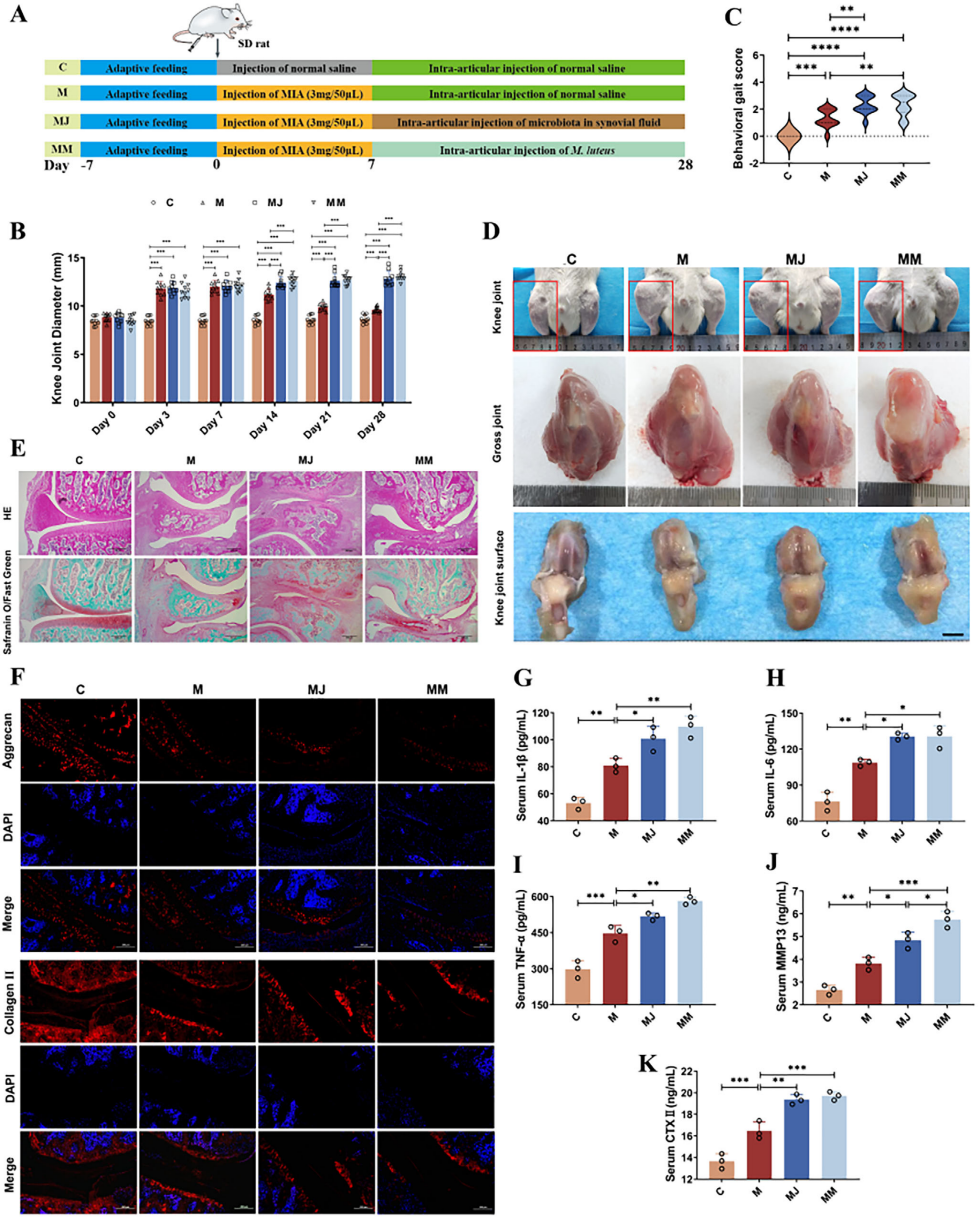
*M. luteus* G18 isolated from OA synovial fluid exacerbates knee OA progression in rats. A) Experimental design. B) Knee joint diameter measurements (n = 10 rats per group). C) Gait scores (n = 10 rats per group). D) Gross morphology and pathological changes of knee joints and articular surfaces. (E) Histological evaluation with H&E and Safranin O–Fast Green staining. F) Immunofluorescence of Aggrecan and Collagen II in cartilage. G–K) Serum concentrations of IL‐1β, IL‐6, TNF‐α, MMP‐13, and CTX‐II. C: Control; M: OA model; MJ: OA synovial fluid intervention; MM: *M. luteus* G18 intervention (n = 3 per group). Data are presented as mean ± SD; statistical analysis by one‐way ANOVA. *p < 0.05, **p < 0.01, ***p < 0.001.

Based on prior analyses integrating synovial fluid high‐throughput sequencing, Spearman correlation analysis and culturomics, *M. luteus* was identified as a potential core microbial strain potentially contributing to the progression of OA. Preliminary experiments further demonstrated that *M. luteus* G18 exhibits pathogenic potential by disrupting chondrocyte homeostasis and reducing chondrocyte viability, likely through the upregulation of pro‐inflammatory cytokine secretion. To validate this hypothesis, a highly active strain of *M. luteus* G18 was selected and intra‐articularly injected into the knee joints of OA‐induced rats to evaluate its impact on OA progression (**Figure** **5**A). Following microbial intervention, the M group rats displayed joint damage consistent with previous observations (Figure 5B–D). Notably, rats in the MM group exhibited exacerbated OA phenotypes, similar to those observed in the MJ group. These included increased gait impairment scores, more pronounced joint swelling, restricted mobility, periarticular tissue hardening, and further narrowing of the joint space (Figure 5B–D). Gross anatomical assessment showed that both MM and MJ groups had markedly more severe joint pathology compared to the M group. This included joint hyperemia, edema, thickened periarticular soft tissues, indistinct joint contours, roughened and discolored articular surfaces, extensive cartilage erosion, and in some areas, full‐thickness cartilage defects exposing the underlying subchondral bone (Figure 5D). Histological examination further confirmed these findings. In the MM group, cartilage tissues exhibited substantial inflammatory cell infiltration, disruption of chondrocyte architecture, and notable loss of ECM—a pattern comparable to the MJ group (Figure 5E). Immunohistochemical and immunofluorescence analyses revealed a significant downregulation of key cartilage structural proteins, including Collagen II and Aggrecan, in the MM group, indicating enhanced cartilage matrix degradation (Figure 5F,G). Consistent with these pathological changes, ELISA assays showed significantly increased levels of pro‐inflammatory cytokines (IL‐1β, IL‐6, TNF‐α) and cartilage catabolic markers (MMP13, CTX‐II) in the serum of MM group rats compared to the M group (*p <* 0.05; Figure 5H–L). These data underscore the pro‐inflammatory and pro‐degradative effects of *M. luteus* G18 within the joint environment.

Taken together, our findings demonstrated that intra‐articular administration of *M. luteus* G18 promotes cartilage destruction, activates inflammatory responses, and accelerates OA progression in rats. These results suggested that *M. luteus* G18 might represent a potential key pathogenic microorganism in the synovial fluid of severe OA patients and a potential target for microbiota‐based OA intervention strategies.

### 2.5 Peptidoglycan from *M. luteus* is the Key Effector Mediating Chondrocyte Damage

**Figure 6:**
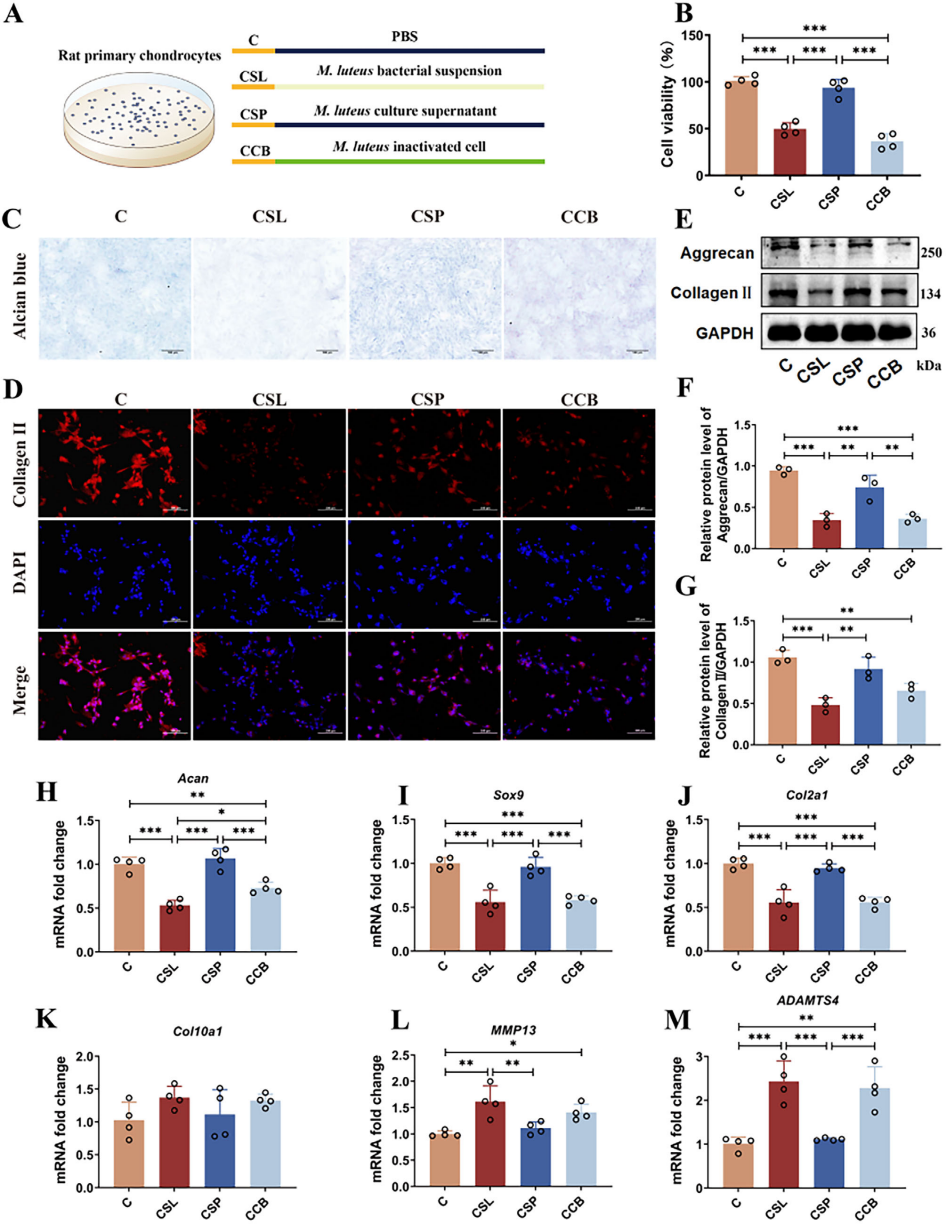
The pathogenic effects of *M. luteus* G18 on knee OA are mediated by its cellular components. A) Schematic of the in vitro experimental design. B) Chondrocyte viability assessed by CCK‐8 assay across treatment groups. C) Alcian blue staining of chondrocytes. D) Immunofluorescence of Collagen II in chondrocytes. E–G) Western blot analysis and relative expression of Aggrecan and Collagen II. (H–M) Relative mRNA expression of chondrogenic anabolic genes (*Acan, Sox9, Col2a1*) and catabolic genes (*Col10a1, Mmp13, Adamts4*). C: Control; CSL: live *M. luteus* G18 suspension; CSP: culture supernatant; CCB: heat‐inactivated bacterial cells (n = 3 per group). Data are presented as mean ± SD; statistical analysis by one‐way ANOVA. *p < 0.05, **p < 0.01, ***p < 0.001.

To elucidate the mechanism by which *M. luteus* G18 aggravates OA by impairing chondrocyte function, we co‐cultured primary rat chondrocytes with live bacteria, bacterial supernatant, and heat‐inactivated *M. luteus* G18. Cell viability of chondrocytes was evaluated through the use of the CCK‐8 assay (**Figure** **6**A). Significant decreases in cell viability were observed in both the CSL and CCB groups relative to the control group (*p <* 0.01; Figure 6B), indicating that *M. luteus* G18—regardless of viability—exerts cytotoxic effects on chondrocytes. In contrast, the bacterial supernatant showed only a mild impact, suggesting that the cell wall components rather than secreted products are responsible for the observed toxicity. Alcian blue staining and immunofluorescence analyses further revealed marked reductions in acidic proteoglycan and Collagen II expression in the CSL and CCB groups (Figure 6C,D), indicating suppressed synthesis of ECM components. Western blot results were consistent with these findings, showing significantly decreased levels of Collagen II and Aggrecan in both groups compared to controls (*p <* 0.01; Figure 6E–G), reinforcing the notion that *M. luteus* G18 impairs cartilage matrix homeostasis. To confirm these results at the transcriptional level, RT‐qPCR was performed to assess gene expression related to cartilage metabolism. Anabolic genes including Acan, Sox9, and Col2a1 were significantly downregulated in both CSL and CCB groups (*p <* 0.01; Figure 6H–J), while catabolic genes such as Col10a1, Mmp13, and Adamts4 were markedly upregulated (Figure 6K–M). These results collectively demonstrate that *M. luteus* G18 disrupts chondrocyte metabolic homeostasis by simultaneously suppressing anabolic pathways and promoting catabolic activity. Notably, the comparable effects observed in both live and inactivated bacteria groups suggest that the key effector is a structural element of the bacterial cell wall.

Given that *M. luteus* G18 is a gram‐positive bacterium, which cell wall is rich in peptidoglycan—a well‐known immunogenic molecule. Previous studies (e.g.*, Kumar* et al.) have shown that bacterial peptidoglycan can induce synovitis in rats and potentially contribute to OA pathogenesis.^[^ ^20^ ^]^ To test whether *M. luteus*‐derived peptidoglycan mediates its deleterious effects on chondrocytes, we incubated primary chondrocytes with purified peptidoglycan at various concentrations (5 , 25 , and 50 µg mL^−1^) and assessed cellular outcomes (Figure S3A, Supporting Information). The CCK‐8 assay revealed a dose‐dependent decrease in chondrocyte viability (*p <* 0.01; Figure S3B, Supporting Information), and Alcian blue staining showed reduced proteoglycan content (Figure S3C, Supporting Information). For subsequent experiments, we selected a moderate concentration (25 µg mL^−1^) approximating the median inhibitory dose. Consistent with previous observations, exposure to peptidoglycan resulted in a marked decrease in the protein expression of Collagen II and Aggrecan (*p <* 0.05; Figure S3D–F, Supporting Information), along with downregulation of anabolic genes (Acan, Sox9, Col2a1) and upregulation of catabolic genes (Col10a1, Mmp13, Adamts4) (*p <* 0.05; Figures S3G–L, Supporting Information).

Collectively, these findings provide strong evidence that peptidoglycan from *M. luteus* G18 is a key pathogenic factor responsible for disrupting chondrocyte metabolic balance, thereby exacerbating cartilage degradation and OA progression. This highlights peptidoglycan as a critical mediator in OA pathophysiology and a potential target for therapeutic intervention.

### 2.6 *M. luteus*‐Derived Peptidoglycan Disrupts Chondrocyte Metabolism via Activation of the TLR2/JNK/AP‐1 Signaling Pathway

**Figure 7:**
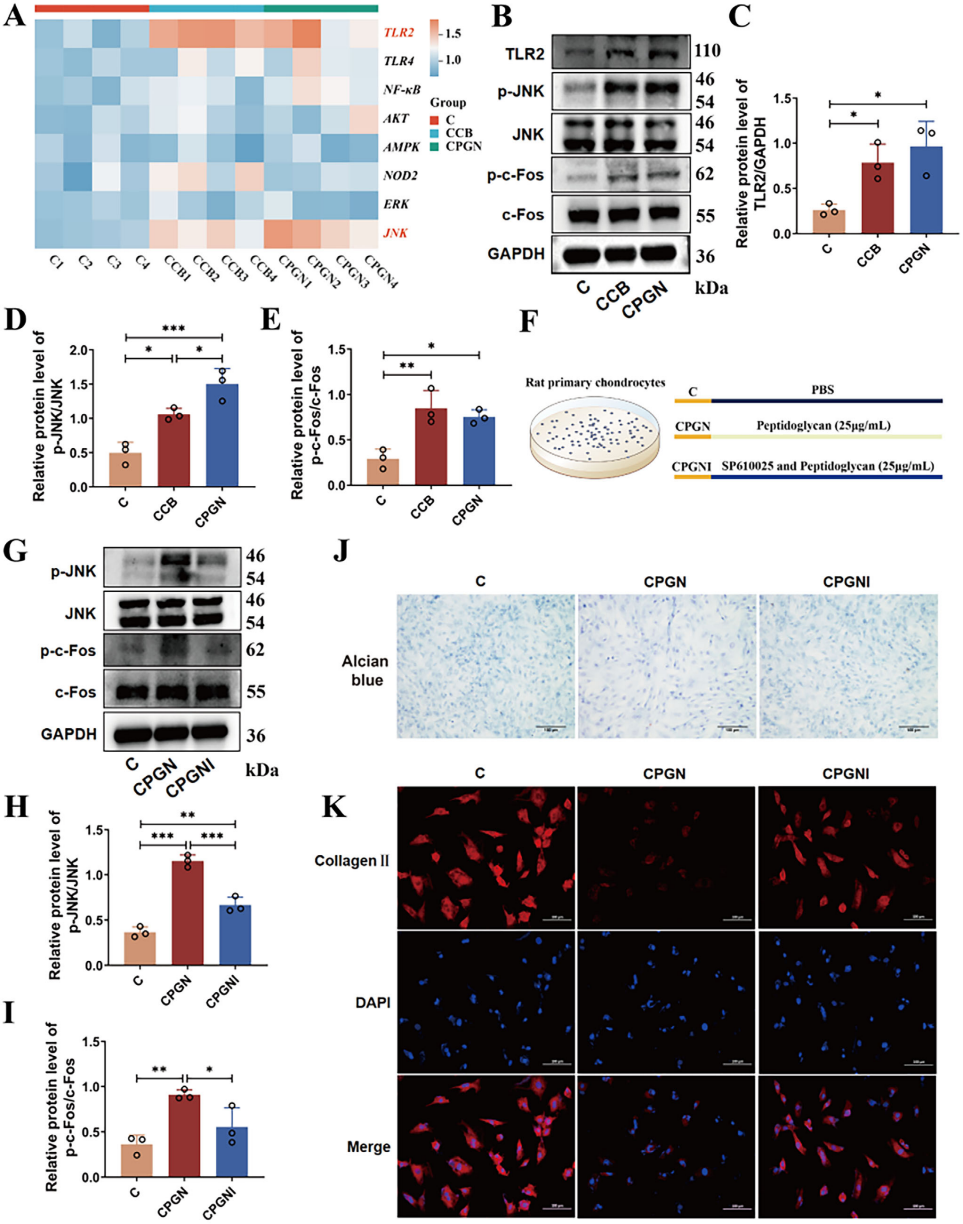
Peptidoglycan from *M. luteus* disrupts chondrocyte metabolism via the TLR2/JNK/AP‐1 signaling pathway. A) Relative mRNA expression of signaling pathway genes associated with chondrocyte damage. B–E) Western blot analysis of TLR2, p‐JNK, JNK, p‐c‐Fos, and c‐Fos in peptidoglycan‐stimulated chondrocytes. F) Schematic of inhibitor intervention experiment. G–I) Protein expression of p‐JNK, JNK, p‐Fos, and Fos following inhibitor treatment. J) Alcian blue staining after inhibitor intervention. K) Immunofluorescence of Collagen II following inhibitor treatment. C: Control; CCB: heat‐inactivated *M. luteus* G18 cells; CPGN: peptidoglycan (25 µg mL^−1^) from *M. luteus*; CPGNI: peptidoglycan + SP600125 (JNK inhibitor) (n = 3 per group). Data are presented as mean ± SD; statistical analysis by one‐way ANOVA. *p < 0.05, **p < 0.01, ***p < 0.001.

In order to clarify the molecular mechanism by which *M. luteus*‐derived peptidoglycan impairs chondrocyte metabolic homeostasis, the expression of pivotal signaling molecules implicated in the recognition of peptidoglycan and its downstream signaling cascades was examined by RT‐qPCR analysis. Relative to the C group, chondrocytes in both the CCB and CPGN groups demonstrated a significant upregulation of the pattern recognition receptor *TLR2* expression (**Figure** **7**A). Among the intracellular signaling molecules tested, *JNK* expression was most notably upregulated (*p <* 0.01), while *AKT* and *NF‐κB* showed mild increases. In contrast, *NOD2*, *ERK*, and *AMPK* levels remained unchanged (Figure 7A). Given that the JNK/AP‐1 (c‐Fos) signaling axis is known to play a central role in OA pathogenesis,^[^ ^21^ ^]^ we hypothesized that *M. luteus* peptidoglycan activates the TLR2 receptor on chondrocyte surfaces, subsequently initiating the JNK/AP‐1 pathway, leading to metabolic disruption. This hypothesis was confirmed by Western blot analysis: both TLR2 protein and the p‐JNK and p‐Fos were significantly increased in the CCB and CPGN groups compared to controls (*p <* 0.05; Figure 7B–E), confirming pathway activation. To verify the causal role of JNK signaling, we employed the JNK‐specific inhibitor SP600125 (Figure 7F). Western blot results demonstrated that SP600125 significantly suppressed the expression of p‐JNK and p‐Fos in the CPGNI group (*p <* 0.05; Figure 7G–I), indicating effective pathway inhibition. Subsequent Alcian blue staining and Collagen II immunofluorescence showed partial restoration of acidic proteoglycans and Collagen II expression in the CPGNI group compared to CPGN alone (Figure 7J,K). In agreement with these findings, Western blot analysis demonstrated increased levels of key structural proteins, Collagen II and Aggrecan, and enhanced chondrocyte viability following JNK inhibition (Figures S4A–D, Supporting Information). RT‐qPCR further confirmed the reversal of peptidoglycan‐induced downregulation of anabolic genes (*Acan, Sox9, Col2a1*) and the attenuation of upregulated catabolic genes (*Col10a1, Mmp13, Adamts4*) in the CPGNI group (Figure S4E–J, Supporting Information).

Collectively, these results demonstrate that *M. luteus*‐derived peptidoglycan disrupts chondrocyte metabolic homeostasis by activating the TLR2/JNK/AP‐1 signaling cascade. This leads to suppression of anabolic gene expression and promotion of catabolic pathways, ultimately accelerating chondrocyte damage. This mechanism likely represents a key molecular basis by which *M. luteus* in joint fluid contributes to OA progression.

### 2.7 Inhibition of the JNK Signaling Pathway Attenuates *M. luteus* G18‐Induced Exacerbation of OA Progression In Vivo

**Figure 8:**
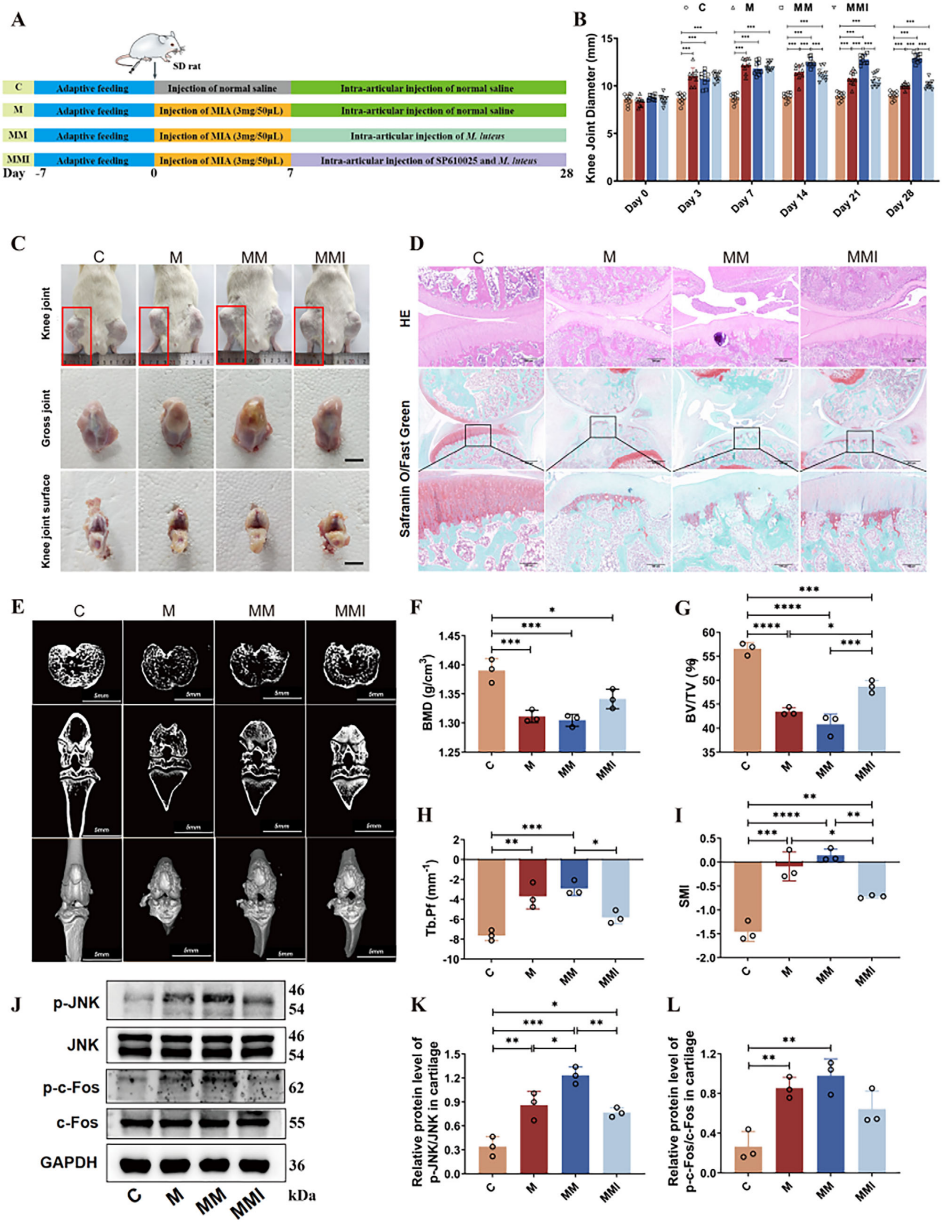
JNK pathway inhibition mitigates the aggravating effect of *M. luteus* G18 on OA progression in rats. A) Experimental design. B) Knee joint diameter measurements (n = 10 rats per group). C) Gross morphology and histopathology of knee joints and cartilage. D) Histological analysis with H&E and Safranin O–Fast Green staining. E) Micro‐CT evaluation of knee joint tissues. F–I) Bone‐related parameters, including bone mineral density (BMD), bone volume fraction (BV/TV), trabecular pattern factor (Tb.Pf), and structural model index (SMI). J–L) Western blot analysis of p‐JNK, JNK, p‐Fos, and Fos expression in cartilage. C: Control; M: OA model; MM: *M. luteus* G18; MMI: *M. luteus* G18 + SP600125 (JNK inhibitor) (n = 3 per group). Data are presented as mean ± SD; statistical analysis by one‐way ANOVA. *p < 0.05, **p < 0.01, ***p < 0.001.

To validate in vivo whether *M. luteus* aggravates OA via activation of the JNK/AP‐1 (c‐Fos) signaling pathway, OA rats treated with *M. luteus* G18 received intra‐articular injections of the selective JNK inhibitor SP600125 (**Figure** **8**A). The intervention significantly mitigated the increase in knee joint diameter and deterioration of gait scores induced by *M. luteus* G18 (*p <* 0.01, Figure 8B, Figure S5A, Supporting Information). Gross examination revealed pronounced joint swelling and redness in the group M, with exacerbated inflammation and cartilage damage in the MM group. In contrast, the MMI group showed reduced joint swelling and improved joint architecture (Figure 8C). Histological analysis using H&E and Safranin O staining confirmed that MMI rats exhibited preserved cartilage architecture, increased GAG content, and maintained extracellular matrix integrity (Figure 8D). Micro‐CT analysis further demonstrated that the M group showed significant bone structural deterioration compared to controls, including reductions in bone mineral density (BMD), bone volume (BV), bone volume fraction (BV/TV), and trabecular cavity CT values (Tb.Cav.CT Value) (*p <* 0.05; Figure 8E–G; Figure S5C,D, Supporting Information). Parameters indicative of trabecular deterioration—trabecular pattern factor (Tb.Pf) and structure model index (SMI)—were significantly increased (*p <* 0.01, Figure 8H,I), suggesting trabecular rod‐like transformation, compromised load‐bearing capacity, and disordered bone remodeling. Moreover, the bone surface‐to‐volume ratio (BS/BV) was elevated in the M group (Figure S5B, Supporting Information), indicating increased bone fragility. These pathological changes were more pronounced in the MM group, whereas the MMI group showed substantial recovery, with values approaching those of the C group, highlighting the therapeutic potential of JNK pathway inhibition. At the molecular level, Western blot analysis revealed significantly elevated expression of p‐JNK and p‐Fos in the M group (*p <* 0.01, Figure 8J–L), along with increased serum levels of pro‐inflammatory cytokines IL‐1β, IL‐6, TNF‐α, and cartilage degradation markers MMP13 and CTX‐II (*p <* 0.01, Figure S4E–I, Supporting Information). These effects were further intensified in the MM group, suggesting persistent activation of the JNK/AP‐1 signaling axis. Notably, SP600125 treatment markedly suppressed the expression of p‐JNK and p‐Fos and reduced circulating inflammatory and catabolic mediators in the MMI group, indicating effective blockade of the pathway and attenuation of cartilage damage.

**Figure 9:**
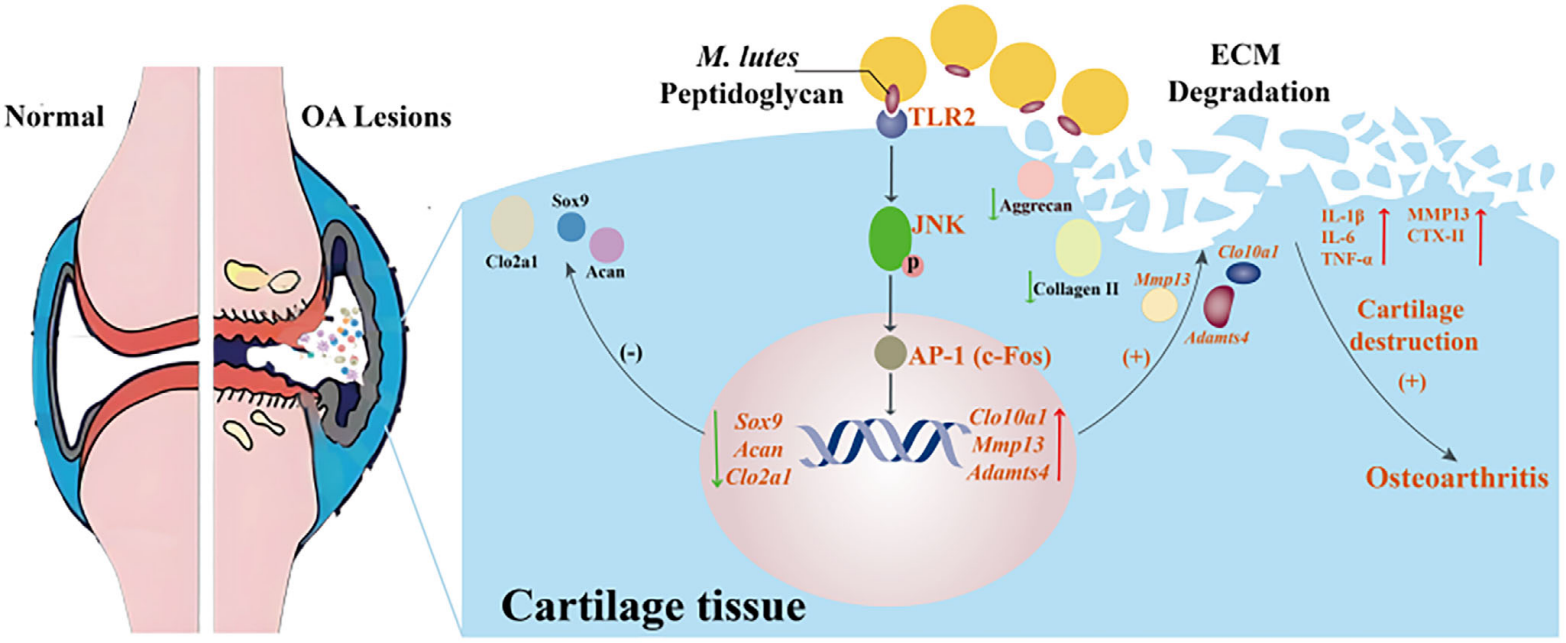
Schematic illustration of the role of synovial *M. luteus* G18 in cartilage degeneration during osteoarthritis.

Taken together, these findings demonstrate that *M. luteus* G18 isolated from the synovial fluid of severe OA patients exacerbates disease progression by activating the JNK/AP‐1 signaling pathway, thereby upregulating pro‐inflammatory and cartilage‐degrading factors (**Figure** **9**). The JNK inhibitor SP600125 effectively reverses these pathological alterations in vivo, confirming the pivotal role of JNK/AP‐1 signaling in *M. luteus* G18‐mediated OA aggravation.

## 3 Discussion

OA represents a prevalent chronic degenerative joint disease, the development of which is driven by a complex interplay of factors such as advancing age, abnormal mechanical stress, obesity, genetic susceptibility, and sustained low‐grade inflammatory responses.^[^ ^22^, ^23^ ^]^ The joint cavity has traditionally been regarded as a sterile environment, and OA‐related inflammation has long been attributed to non‐infectious immune activation.^[^ ^24^ ^]^ However, recent progress in omics approaches, especially metagenomic analysis and 16S rRNA high‐throughput sequencing, has uncovered bacterial DNA within synovial fluid and joint tissues.^[^ ^25^, ^26^ ^]^ These findings contest the traditional view of joints as sterile environments. Despite these discoveries, whether the detected low‐abundance microbial signals reflect a resident and stable intra‐articular microbiome or transient contamination by exogenous microorganisms remains unresolved. Critical questions persist regarding the existence, stability, and composition of microbial communities within the joint cavity, the identity and origin of specific taxa, and their potential roles—either direct or indirect—in OA development and progression. Clarifying these aspects represents a significant challenge and priority within the current OA research landscape.

In this study, SEM revealed the presence of numerous rod‐shaped and coccoid bacteria in synovial fluid (Figure 1B), challenging the traditional concept of the joint cavity as a strictly “sterile” environment. Further 16S rRNA high‐throughput sequencing analysis demonstrated a diverse microbial community within the synovial fluid. At the phylum level, the microbial composition was dominated by Proteobacteria, Firmicutes, Actinobacteria, and Bacteroidetes (Figure 1F), consistent with previous reports by *Zhao* et al.^[^ ^27^ ^]^ Genus‐level analysis identified *Pseudomonas*, *Lactobacillus*, and *Cupriavidus* as predominant taxa (Figure 1G). Moreover, *Tsai* et al. reported detectable low‐abundance microbes even in the synovial fluid of non‐OA subjects, further supporting the notion that the joint cavity may not be absolutely sterile.^[^ ^28^ ^]^ Comparative analysis of synovial microbiota across OA patients with varying disease severities demonstrated a significant increase in α‐diversity in severe OA cases, accompanied by marked differences in β‐diversity profiles (Figure 1C,D), suggesting a dynamic restructuring of the microbial community during OA progression. In the current field of microbiome research, higher levels of gut microbial diversity are generally associated with the maintenance of host homeostasis.^[^ ^29^ ^]^ In contrast, increased vaginal microbial diversity is often linked to adverse health outcomes, such as infection and inflammatory responses.^[^ ^30^ ^]^ These observations suggest that microbial ecosystems in different anatomical sites have fundamentally distinct functional requirements for diversity. However, regarding the hypothesis that “elevated synovial fluid microbial diversity necessarily leads to impaired joint health,” there is currently insufficient evidence to support this assertion. This proposition requires further validation through larger clinical cohorts and more in‐depth mechanistic studies, such as investigations into microbe–host interaction pathways. Notably, the relative abundances of potentially pathogenic genera such as *Cupriavidus*, *Comamonas*, *Micrococcus*, and *Prevotella* were significantly elevated in patients with severe OA (Figure 1I–L, Figure S1M, Supporting Information). Evidence from Fernández‐Rodríguez et al. supports these observations, reporting enhanced microbial diversity in synovial fluid from OA‐affected knees compared to non‐OA controls.^[^ ^25^ ^]^ This pattern suggests a potential association between elevated microbial diversity and the development or progression of osteoarthritis. Correlation analysis integrating sequencing data with clinical staging further identified genera including Acinetobacter, Brevundimonas, Arthrobacter, Propionibacterium, Prevotella, and Micrococcus whose relative abundances positively correlated with OA severity and disease duration (Figure 2A). These findings align with *Tsai* et al*.’s* hypothesis that dysbiosis within the joint microbiome may contribute to OA progression, likely through modulation of local inflammatory responses or disruption of cartilage homeostasis.^[^ ^28^ ^]^ Taken together, the data provide compelling evidence that distinct synovial microbial communities contribute to the underlying pathophysiological mechanisms involved in osteoarthritis progression.

Furthermore, synovial fluid samples from OA patients were subjected to culturomics analysis, resulting in the isolation of over 145 bacterial strains representing 57 distinct species, differentiated by colony morphology, color, and size. Notably, *M. luteus* emerged as the most frequently isolated species (with 26 strains identified), of which 23 were derived from the synovial fluid of patients with severe OA (Figure 2). This predominance suggests a potentially significant role for *M. luteus* within the joint microecology of OA. *M. luteus* is a gram‐positive coccus ubiquitously distributed on human skin, oral mucosa, and in natural environments,^[^ ^31^, ^32^ ^]^ which originally described by the German microbiologist Joseph Schröter and subsequently named by Cohn for its characteristic yellow colonies.^[^ ^33^ ^]^ Interestingly, recent studies have increasingly reported the presence of *M. luteus* in traditionally sterile sites such as blood and cerebrospinal fluid, where its cell wall PGN is capable of activating host immune responses and inducing pro‐inflammatory cytokines, including IL‐6 and TNF‐α.^[^ ^34^, ^35^, ^36^ ^]^ These findings imply that *M. luteus*’ ecological role extends beyond that of a mere commensal or contaminant. Although traditionally regarded as an opportunistic pathogen, *M. luteus* is primarily associated with bacteremia (incidence ≈3.5%) and catheter‐associated biofilm infections, particularly in immunocompromised individuals.^[^ ^37^ ^]^ For instance, *Ianniello* et al. documented *M. luteus* involvement in infective endocarditis in patients with non‐Hodgkin lymphoma,^[^ ^38^ ^]^ while *Pederson* et al. reported a fatal sepsis case caused by *M. luteus* in an immunodeficient patient.^[^ ^39^, ^40^ ^]^ Moreover, metagenomic analyses further revealed that *M. luteus* harbors *mecA* gene variants conferring potential antibiotic resistance, alongside adhesin genes such as fnbA and hemolysin‐like virulence factors, which may enhance tissue invasiveness and pathogenicity.^[^ ^37^ ^]^ Importantly, our preliminary in vitro experiments demonstrated that *M. luteus* triggers elevated inflammatory responses and disrupts anabolic metabolism in chondrocytes, confirming its capacity to induce pro‐inflammatory and tissue‐destructive effects. Taken together, considering its high isolation frequency in OA synovial fluid and these pathogenic attributes, *M. luteus* likely serves as a potential key “microecological driver” in OA pathogenesis.

To explore the contributory role of synovial fluid microbiota in the initiation and advancement of osteoarthritis, microbial transplantation was conducted in a MIA‐induced rat model of OA (Figure 3A). The results demonstrated that transplantation of microbiota from the S group significantly exacerbated cartilage destruction in recipient rats, characterized by disorganized cartilage matrix structure, abnormal chondrocyte arrangement, and markedly reduced GAG content. These histopathological alterations were associated with a marked reduction in the expression of essential ECM components of cartilage, specifically Aggrecan and Collagen type II (Figure 3). As fundamental constituents of the cartilage ECM, Aggrecan and Collagen II collaboratively maintain cartilage biomechanical properties and metabolic homeostasis, and their decreased expression indicates compromised cartilage integrity.^[^ ^41^, ^42^ ^]^ Moreover, transplantation of synovial fluid microbiota further increased the microbial diversity and the abundance of pathogenic bacteria within the joint cavity of arthritic rats, thereby exacerbating the disruption of the intra‐articular microenvironment and accelerating the progression of OA (Figure 4). Subsequently, we conducted monocolonization experiments with *M. luteus* G18—the most frequently isolated species in the S group—and observed similar exacerbations in cartilage damage, including reduced matrix staining intensity, disrupted cartilage architecture, and diminished expression of critical ECM proteins (Figure 5), implicating *M. luteus* G18 as a putative pathogenic microorganism in OA progression. Mechanistic investigation through in vitro co‐culture of primary chondrocytes with *M. luteus* G18 revealed that PGN, a principal component of the gram‐positive bacterial cell wall, disrupted chondrocyte metabolic homeostasis. PGN exposure suppressed the level of anabolic genes, including Acan, Col2a1, and Sox9, while simultaneously upregulating catabolic mediators such as Mmp13, Adamts4, and Col10a1 (Figure 6, Figure S3, Supporting Information). The imbalance of cartilage metabolism is a hallmark of OA pathophysiology. Aggrecan, encoded by Acan, is the major cartilage proteoglycan responsible for water retention and compressive resilience.^[^ ^43^ ^]^ Collagen II, encoded by Col2a1, provides the 3D framework essential for cartilage structure and function.^[^ ^44^ ^]^ Sox9 is a master transcription factor in chondrogenesis, directly activating *Col2a1* transcription to promote type II collagen synthesis.^[^ ^45^ ^]^ In contrast, Mmp13 encodes a collagenase that selectively degrades Collagen II, serving as a key effector of ECM breakdown.^[^ ^46^ ^]^ Moreover, elevated expression of *Adamts4* and *Col10a1* synergistically accelerates Aggrecan degradation, further promoting cartilage degeneration.^[^ ^45^ ^]^ RT‐qPCR analysis of key signaling molecules revealed that *M. luteus*‐derived PGN activates TLR2 on chondrocyte surfaces, subsequently inducing downstream activation of the JNK/AP‐1 (c‐Fos) signaling pathway (Figure 7A–E). TLR2, an innate immune receptor recognizing gram‐positive bacterial PGN, mediates pathogen‐associated molecular pattern (PAMP) recognition and immune response initiation.^[^ ^47^ ^]^ The JNK pathway is a component of the MAPK family, regulates chondrocyte apoptosis, inflammation, and matrix‐degrading enzyme expression, which has been identified as a critical mediator in OA pathogenesis.^[^ ^48^ ^]^ Additionally, *Han* et al. demonstrated that JNK/AP‐1 signaling promotes Mmp13 transcription, thereby accelerating matrix degradation and cartilage loss.^[^ ^49^ ^]^ To validate the pivotal role of the TLR2/JNK/AP‐1 pathway in *M. luteus* G18‐mediated pathogenicity, we applied the JNK inhibitor SP600125 in both in vivo and in vitro models. JNK inhibition significantly ameliorated *M. luteus* G18‐induced cartilage damage, evidenced by restored cartilage architecture, increased GAG content, and recovery of Aggrecan and Collagen II expression, alongside marked downregulation of inflammatory mediators and matrix‐degrading enzymes (Figure 8, Figure S5, Supporting Information). Collectively, these findings provide compelling evidence that *M. luteus* G18 contributes to OA progression by disrupting chondrocyte metabolic equilibrium through TLR2‐mediated activation of the JNK/AP‐1 signaling pathway. This work identifies *M. luteus* G18 as a potential pathogenic driver in OA and underscores the pathophysiological relevance of the joint microbiome in modulating disease severity.

This study proposes a novel concept with important implications for understanding synovial fluid microbiota and joint health, potentially informing the clinical management of OA. Nonetheless, several limitations should be noted. The clinical cohort was relatively small, which may limit statistical power and generalizability. Moreover, the culturomics conditions used in this study were suboptimal, which may have limited the isolation of certain synovial fluid microorganisms. Mechanistic analyses focused primarily on inflammatory signaling, particularly the JNK pathway, without fully addressing innate immune cell infiltration, cytokine networks, or broader microbe–host interactions. Future studies will expand clinical validation through larger, multi‐center cohorts, refine microbial isolation and culture strategies and further investigate multidimensional microbe–host interactions—including immune regulation, cytokine signaling, and metabolic pathways—with the goal of constructing a comprehensive “microbiota–immune–inflammation” regulatory network. These efforts are expected to enhance the understanding of synovial fluid microbiota in joint health and provide a clear direction for subsequent research.

## 4 Conclusions

This investigation systematically provides the first robust evidence for the authentic presence and community structure of microbiota within the human knee joint cavity, thereby challenging the longstanding paradigm that the joint space is a sterile environment. By integrating 16S rRNA high‐throughput sequencing with culturomics, a diverse and relatively stable microbial community was identified, predominantly composed of the phylum Actinobacteria, with Micrococcus emerging as the dominant genus. These findings imply that certain microbial taxa are capable of persistent colonization within the local joint microenvironment. Further investigations pinpointed *M. luteus* G18 as a key microorganism closely associated with OA progression. Both in vivo microbial transplantation and in vitro chondrocyte co‐culture experiments demonstrated that *M. luteus* G18 exacerbates OA pathogenesis through its cell wall component PGN, which activates the TLR2/JNK/AP‐1 signaling pathway. Activation of this pathway disrupts chondrocyte metabolic homeostasis by perturbing the anabolic‐catabolic balance, thereby promoting ECM degradation and inflammatory responses (Figure 9). Collectively, these findings not only comprehensively characterize the synovial microbiome in OA patients but also establish *M. luteus* G18 as a potential pathogenic driver in OA progression. This research presents new perspectives on the etiology of OA by elucidating the role of microbe‐host interactions, and establishes a theoretical framework that may support the advancement of microbiota‐informed diagnostic methodologies and precision‐targeted therapeutic approaches for OA management.

## 5 Experimental Section

### Participant Enrollment and Synovial Fluid Microbiota Profiling

A cohort of 40 individuals with clinically confirmed non‐infectious knee osteoarthritis was recruited from the Second Affiliated Hospital of Nanchang University to investigate the synovial fluid microbial composition. Classification of patients was performed based on the Kellgren‐Lawrence (KL) grading criteria, resulting in two distinct groups: the severe osteoarthritis cohort (S group), comprising individuals with KL grades III to IV (n = 20), and the mild osteoarthritis cohort (M group), including those with KL grades I to II (n = 20) Inclusion criteria were as follows: (1) clinically and radiologically confirmed diagnosis of knee OA (via X‐ray or MRI); (2) age between 40 and 75 years; and (3) absence of other serious joint diseases, such as rheumatoid arthritis or gouty arthritis. Exclusion criteria included: (1) acute knee joint injuries (e.g., ligament tears, meniscal injuries); (2) systemic diseases such as hepatic or renal dysfunction, or malignancies; (3) current antibiotic therapy; and (4) history of infectious arthritis. Synovial fluid samples ranging from 2 to 5 mL were aseptically aspirated from the knee joint cavities of all enrolled subjects via arthrocentesis conducted under strictly sterile conditions. The detection of microbiota within synovial fluid was initially established by scanning electron microscopy. Subsequent analysis of the microbial community structure was performed using high‐throughput sequencing. Additionally, key bacterial strains were isolated and identified through culturomics techniques. Written informed consent was obtained from each participant before the collection of biological samples.

### Rats and Treatments

A total of 120 male SD rats (aged 6–8 weeks) were obtained from SPF (Beijing) Biotechnology Co., Ltd. The animals were maintained in a specific pathogen‐free (SPF) facility under controlled environmental conditions, including a temperature of 23 ± 3 °C, humidity of 51 ± 13%, and a 12‐h light/dark cycle. OA was experimentally induced by administering a single intra‐articular injection of 3 mg monosodium iodoacetate (MIA; Sigma‐Aldrich, Batch No. SLBZ7569; St. Louis, MO, USA) dissolved in 50 µL volume into the right knee joint.^[^ ^50^ ^]^ To investigate the impact of synovial fluid microbiota from patients with severe OA on disease progression, 40 SD rats were randomly divided into four groups (n = 10 per group) and subjected to the following interventions: C group: intra‐articular injections of 50 µL sterile saline were administered into the knee joint cavity twice a week; M group: OA was induced via intra‐articular injection of MIA, followed by twice‐weekly intra‐articular administration of 50 µL sterile saline; MA group: pretreatment with a broad‐spectrum antibiotic cocktail (1 g L^−1^ ampicillin, 0.5 g L^−1^ vancomycin, 1 g L^−1^ neomycin, 1 g L^−1^ metronidazole) administered via drinking water for 2 weeks, an OA model was established by means of intra‐articular injection of MIA, followed by twice‐weekly intra‐articular administration of 50 µL sterile saline; MAJ group: following the same antibiotic pretreatment and OA induction protocol as the MA group, intra‐articular injections of 50 µL synovial fluid microbiota collected from patients with severe OA (S group) twice a week, starting on day 7. Knee joint diameter, swelling, joint space, and mobility were monitored. After 4 weeks, rats were euthanized, and serum, knee joint tissues, and synovial lavage fluid were collected for further analysis. To evaluate the specific role of *M. luteus* G18 isolated from the synovial fluid of S group, an additional 40 SD rats were randomly allocated into four experimental groups (n = 10 per group): C group: intra‐articular injection of 50 µL sterile saline twice a week; M group: MIA‐induced OA followed by intra‐articular injection of 50 µL sterile saline twice a week; MJ group: MIA‐induced OA followed by intra‐articular injection of 50 µL synovial fluid microbiota from the S group twice a week; MM group: MIA‐induced OA followed by intra‐articular injection of 1 × 10⁴ CFU/50 µL *M. luteus* G18 twice a week. As described above, changes in the rat knee joints were monitored over a 4‐week period, after which joint tissue samples were collected for further analysis. To assess whether *M. luteus* G18 promotes OA progression via the JNK/AP‐1 signaling pathway, 40 additional rats were randomly divided into four groups (n = 10 per group): C group: intra‐articular injection of 50 µL sterile saline twice a week; M group: MIA‐induced OA followed by intra‐articular injection of 50 µL sterile saline twice a week; MM group: MIA‐induced OA followed by intra‐articular injection of 1 × 10⁴ CFU/50 µL *M. luteus* G18 twice a week; MMI group: MIA‐induced OA followed by intra‐articular injection of *M. luteus* G18 (1 × 10⁴ CFU/50 µL) and the JNK inhibitor SP600125 (10 mg kg^−1^) twice a week. As described above, changes in the rat knee joints were monitored over a 4‐week period, after which joint tissue samples were collected for further analysis. Quantitative evaluation of knee joint swelling was performed using a calibrated digital caliper to measure the diameter of the right knee at designated time points: days 0, 1, 3, 7, 14, 21, and 28. 12 h following the final intervention, gait assessment was performed by applying black ink to the plantar surfaces of the hind limbs. Each animal was then permitted to ambulate across a white paper walkway to obtain footprints for gait pattern evaluation. The gait of the injected (right) limb was visually compared with the non‐injected (left) limb to assess weight‐bearing capacity.^[^ ^20^ ^]^ Gait scores were assigned according to the criteria in Table S4, Supporting Information. The quantification of serum IL‐1β, IL‐6, TNF‐α, MMP13, and CTX‐II was conducted by ELISA kits. Histological evaluation of articular cartilage morphology was performed through H&E staining. Safranin O–fast green staining was employed to evaluate glycosaminoglycan content and distribution in the extracellular matrix. Immunofluorescence staining was applied to examine the expression levels of key cartilage matrix proteins, Collagen II and Aggrecan. TUNEL staining was conducted to detect chondrocyte apoptosis in joint cartilage samples.

### Cell Culture and Stimulation

Primary rat articular chondrocytes (RAT‐AWCell‐s003) were obtained from Cyagen Biosciences Inc. (Shanghai, China) and cultured using the iCell primary chondrocyte culture system (Catalog No. PriMed‐iCELL‐020). Cell cultures were incubated at 37 °C in a humidified atmosphere containing 5% carbon dioxide to ensure optimal growth conditions. The culture system was composed of chondrocyte basal medium supplemented with 1% proprietary chondrocyte culture additives, 10% fetal bovine serum (FBS), 100 units mL^−1^ penicillin, and 100 µg mL^−1^ streptomycin to support cell viability and maintain aseptic conditions. Upon reaching ≈95% confluence, chondrocytes were plated at a density of 5 × 10⁶ cells per well in 6‐well culture plates preloaded with sterile glass coverslips, followed by incubation under standard conditions for 12–16 h to allow cell adherence prior to experimental intervention. To investigate the key components of *M. luteus* G18 responsible for chondrocyte damage, cells in the logarithmic growth phase were divided into the following groups: C group: treated with phosphate‐buffered saline (PBS); CSL group: Treated with 10⁴ CFU of *M. luteus* G18 cultured for 24 h; CSP group: treated with 24‐h bacterial culture supernatant; CCB group: treated with *M. luteus* G18 inactivated by heating at 100 °C for 15 min.^[^ ^51^ ^]^ Following a 4‐h incubation with the designated treatments at 37 °C, cultured cells were harvested for subsequent molecular analyses.^[^ ^52^ ^]^ To determine whether *M. luteus* G18 impairs chondrocyte metabolism via its peptidoglycan (PGN), logarithmic‐phase chondrocytes were grouped and treated as follows: C group: Treated with PBS; CCB group: treated with heat‐inactivated *M. luteus* G18 (24‐h culture); CPGN group: treated with purified *M. luteus* peptidoglycan (Sigma, 10 mg, MFCD00212486) at concentrations of 5, 25, or 50 µg mL^−1^. All experimental treatments were applied with incubation at 37 °C for a duration of 4 h, after which cells were collected for molecular evaluation. To further elucidate whether *M. luteus* peptidoglycan disrupts chondrocyte metabolism via the JNK/AP‐1 signaling pathway, logarithmic‐phase cells were treated under the following conditions: C group: treated with PBS; CPGN group: treated with 25 µg mL^−1^ *M. luteus* peptidoglycan; CPGNI group: treated with 25 µg mL^−1^ *M. luteus* peptidoglycan plus 10 µmol L^−1^ of the JNK inhibitor SP600125. Cell cultures were maintained at 37 °C for a period of 4 h before harvesting for subsequent molecular analyses. Cellular proliferation was evaluated utilizing the CCK‐8 assay according to the manufacturer's protocol. Acidic glycosaminoglycan (GAG) content—an indicator of extracellular matrix integrity—was evaluated using Alcian Blue staining to assess chondrocyte matrix degradation, particularly levels of chondroitin sulfate and keratan sulfate.

### Scanning Electron Microscopy

Synovial fluid specimens underwent initial centrifugation at 10000 × g for 10 min to separate the cellular fraction from the supernatant. The resulting cell pellet was subjected to three sequential washes with PBS, each followed by centrifugation at 10000 × g for 5 min to ensure thorough removal of residual supernatant. Subsequently, the purified cell pellet was fixed in 2.5% glutaraldehyde at 4 °C for 2 h. Post‐fixation, samples were dehydrated through a graded ethanol series consisting of 30%, 50%, 70%, 90%, and 100% ethanol concentrations, with each step lasting 10 min. Dehydrated samples were subjected to critical point drying using a Hitachi HCP‐2 critical point dryer (Hitachi, Japan). The dried samples were then mounted on aluminum stubs and coated with a ≈10 nm thick gold film using an ion sputter coater (Hitachi E‐1010, Japan). Subsequent examination of the prepared samples was performed by scanning electron microscope (Hitachi S‐4800, Japan) operated at an accelerating voltage of 3.0 kV. Images were captured at magnifications ranging from 5000× to 50000× to observe the morphology and spatial distribution of bacteria present in the synovial fluid.

### Microbiome Analysis

Total DNA was extracted from synovial fluid samples. The V3–V4 hypervariable region of the 16S rRNA gene was amplified using universal bacterial primers (forward: 5′‐GTGYCAGCMGCCGCGGTAA‐3′; reverse: 5′‐GGACTACNVGGGTWTCTAAT‐3′) on the Illumina platform. Raw sequencing reads were quality‐filtered and trimmed using Vsearch (v2.13.4) and Cutadapt (v2.3) to remove adapters, primers, and low‐quality sequences. Amplicon sequence variants (ASVs) were then generated, and the length distribution of high‐quality sequences was assessed. Taxonomic assignment was performed using the classify‐sklearn algorithm in QIIME2 (2019.4), referencing Greengenes, NT, and UNITE databases through a pretrained naïve Bayes classifier, covering taxonomic ranks from kingdom to species. For community analyses, principal coordinate analysis (PCoA) was conducted using QIIME2 and R packages, while non‐metric multidimensional scaling (NMDS) was performed with the vegan package. Venn diagrams were generated using the VennDiagram package. Normality of clinical cohort variables was assessed with the Shapiro‐Wilk test. Continuous variables were presented as mean ± standard deviation if normally distributed or as median and interquartile range (IQR) if non‐normal. Categorical variables were expressed as counts and percentages. Group comparisons employed the chi‐square test for categorical variables, independent‐sample t‐tests for normally distributed continuous variables, and Mann–Whitney U tests for non‐normal data. Multi‐group experimental comparisons were analyzed using one‐way ANOVA with Tukey's post hoc test. Statistical significance was set at two‐tailed *p <* 0.05. All raw sequencing data generated in this study have been deposited in the NCBI Sequence Read Archive (SRA) under accession number PRJNA1288057 and are publicly accessible.

### Culturomics

Synovial fluid samples (2–5 mL) were first preprocessed and diluted: after centrifugation at 500 × g for 5 min to remove synovial cells and tissue debris, the supernatant was serially diluted in sterile saline (10⁰, 10^−1^, 10^−2^). This step was designed to prevent overgrowth in high‐density samples while ensuring that low‐abundance microbes could form visible colonies for single‐colony isolation. Diluted samples were then plated on four types of solid media (BHI, BHI supplemented with sheep blood, BHI supplemented with skim milk, and LB) and incubated at 37 °C under both aerobic and anaerobic conditions for 24–72 h, thereby capturing both fast‐growing aerobes and slow‐growing anaerobes. Colonies were selected based on morphological characteristics and subsequently identified by 16S rDNA sequencing. The choice of media was made to maximize microbial diversity: BHI supports most non‐fastidious bacteria; BHI with sheep blood provides additional growth factors for fastidious bacteria (e.g., some Streptococcus spp.) and allows preliminary differentiation by hemolysis patterns; BHI with skim milk enhances growth of microbes utilizing complex nutrients; and LB serves as a reproducible control medium to ensure reliability of cultivation results.

### Western Blotting

Proteins were extracted from homogenized cartilage tissue and cultured chondrocytes using RIPA lysis buffer supplemented with a protease inhibitor cocktail, ensuring preservation of protein integrity for subsequent analyses. Following lysis, the resulting protein mixtures were centrifuged at 12 000 rpm for 20 min at 4 °C to remove cellular debris. The separation of the protein of interest was then conducted via SDS‐PAGE, utilising polyacrylamide gels with a range of 8–15% purity. Following electrophoresis, proteins were transferred onto polyvinylidene difluoride (PVDF) membranes (Millipore, Boston, MA, USA; IPVH00010). To reduce nonspecific binding, membranes were blocked at room temperature for 90 min using a standard blocking solution. Subsequently, membranes were incubated with specific primary antibodies at 4 °C overnight to allow for targeted antigen recognition. After thorough washing with Tris‐buffered saline containing Tween‐20 (TBST), membranes were incubated with appropriate horseradish peroxidase‐conjugated secondary antibodies for 1 h at room temperature. Detection of immunoreactive protein bands was achieved using an enhanced chemiluminescence (ECL) reagent (Thermo Fisher Scientific, Waltham, MA, USA; 32 209). Signal intensity was quantified using ImageJ software to facilitate downstream analysis.

### RNA Extraction and Quantitative Real‐Time PCR

Total RNA was extracted from primary rat chondrocytes using TRIzol reagent following the manufacturer's instructions. RNA concentration and purity were assessed with a NanoDrop 2000 spectrophotometer. Complementary DNA (cDNA) was synthesized using the PrimeScript RT kit, with gDNA Eraser applied to remove any genomic DNA contamination. Quantitative real‐time PCR (qRT‐PCR) was performed on the ViiA7 system using the following cycling program: initial denaturation at 95 °C for 5 min, followed by 40 cycles of 95 °C for 10 s, 60 °C for 20 s (annealing), and 72 °C for 20 s (extension). Gene expression levels were quantified using the 2^−ΔΔCt^ method, and detailed primer sequences are provided in Table S6, Supporting Information.

### Micro‐CT Analysis

Micro‐CT was employed to assess osteophyte formation and subchondral bone remodeling within rat knee joints. Following euthanasia, intact knee joints (with skin and muscle tissues removed) were harvested for analysis. Specimens (n = 3 per experimental group) underwent scanning with a Siemens Inveon MM Gantry micro‐CT system at an isotropic voxel resolution of 5 µm^3^. Quantitative analysis was performed using the Inveon Research Workplace software to evaluate key parameters reflecting bone microarchitecture and structural integrity.

### Statistical Analysis

Statistical analyses were performed utilizing GraphPad Prism version 9 (GraphPad Software, San Diego, CA, USA) and IMB SPSS Statistics 26 (IBM Corp., Armonk, NY, USA). The normality of variable distributions in the clinical cohort was assessed by the Shapiro‐Wilk test. Normally distributed continuous data were presented as mean ± standard deviation (SD), whereas variables with non‐normal distribution were expressed as median and interquartile range (IQR). Categorical variables were summarized as counts and percentages. Between‐group comparisons were performed using the chi‐square (χ^2^) test for categorical variables, the independent‐samples t‐test for normally distributed continuous variables. For continuous variables lacking normality, the Mann‐Whitney U test was applied. Spearman correlation analysis was employed to examine the associations between clinical parameters and key differential microbial taxa, utilizing the corrplot package in R version 4.4.0. Experimental data involving multiple groups were analyzed by one‐way analysis of variance (ANOVA) followed by Tukey's post hoc test for pairwise comparisons. Statistical significance was defined as a two‐tailed *p <* 0.05.

### Ethics

Ethical approval for the study was granted by the Biomedical Research Ethics Committee of the Second Affiliated Hospital of Nanchang University (Approval No. 2023. 25). All experimental protocols involving animals were approved by the Institutional Animal Care and Use Committee of Nanchang University (Approval No. NCULAE‐20221226001).

## Conflict of Interest

The authors declare no conflict of interest.

## Author Contributions

T.‐T.C. and Q.‐W.Z. contributed equally to this work. L.H. and T.‐T.C. oversaw and managed the overall progress of the project. T.‐T.C. and Q.‐W.Z. contributed equally to experiment design and implementation. T.‐C.X. and X.‐Y.Q. assisted with experimental procedures. K.‐Y.L. and J.W. organized the data and compiled the results. L.H., T.‐T.C., and Q.‐W.Z. carried out data analysis and prepared the manuscript. All authors reviewed and approved the final manuscript.

## Supporting information



Supporting Information

## Data Availability

The complete raw data generated in this study have been submitted to the National Center for Biotechnology Information (NCBI) Sequence Read Archive (SRA) and are publicly accessible under the accession number PRJNA1288057.
