# Supplementary material for: Synovial Fluid‐derived Micrococcus Luteus G18 Exacerbates Osteoarthritis Progression by Promoting Chondrocyte Degradation via TLR2/JNK/AP‐1 Signaling Pathway
Source: Adv Sci (Weinh). 2025 Nov 16;13(6):e14220. doi: 10.1002/advs.202514220 (PMC12866835; doi:10.1002/advs.202514220)
Supplement: Supplementary file 1 — Supporting Information [file ADVS-13-e14220-s001.docx]

**Supplementary Materials**


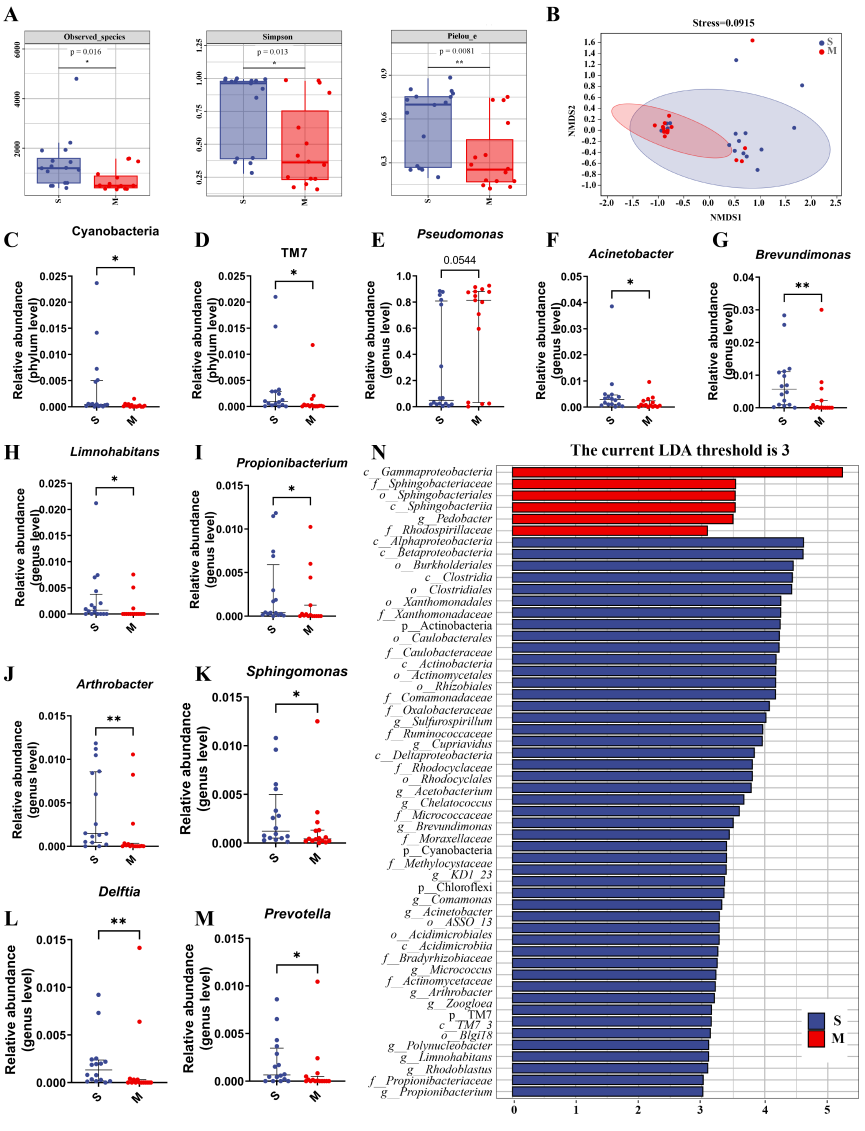


**Figure S1. Microbial composition and differential analysis of synovial fluid in OA patients.** (A) Alpha diversity analysis. (B) Beta diversity analysis. (C, D) Phylum-level relative abundance of Cyanobacteria and TM7. (E–M) Genus-level comparative abundances of Pseudomonas, Acinetobacter, Brevundimonas, Limnohabitans, Propionibacterium, Arthrobacter, Sphingomonas, Delftia, and Prevotella. (N) LEfSe-derived taxonomic cladogram; circle size reflects relative abundance. S: Severe knee OA; M: Mild knee OA. Data are presented as median (IQR); statistical analysis by Mann–Whitney U test. *p < 0.05, **p < 0.01, ***p < 0.001.


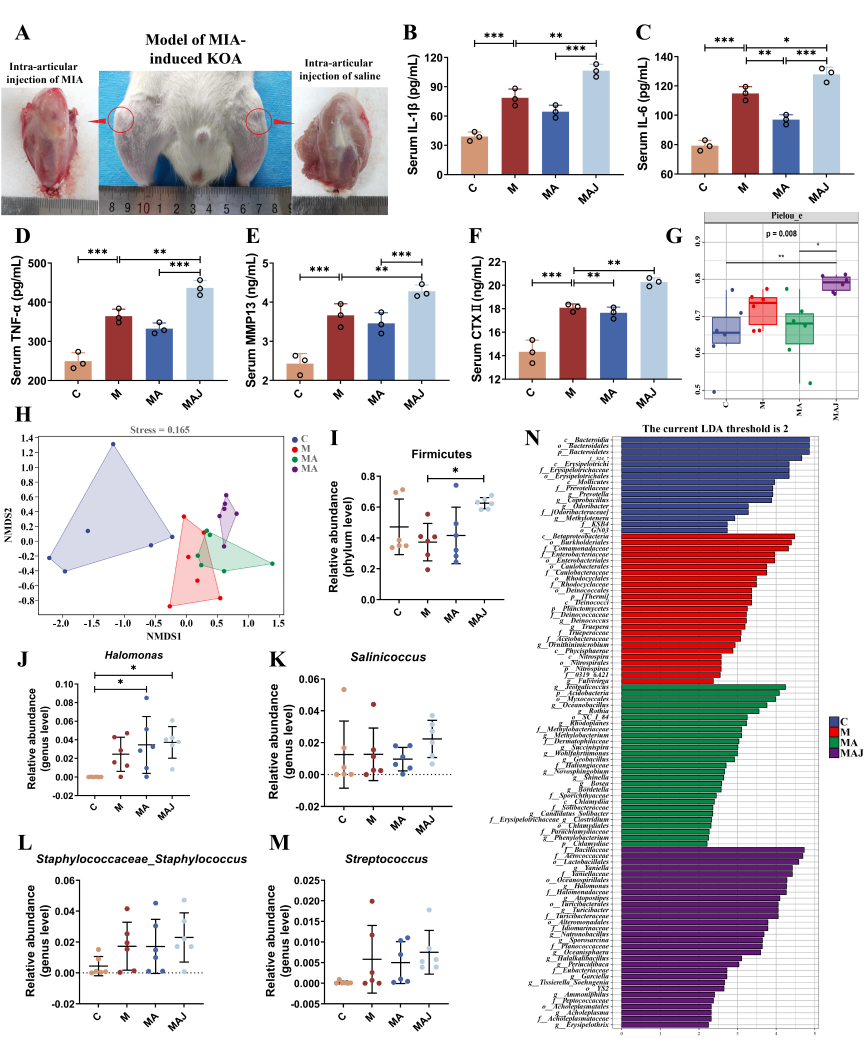


**Figure S2. Serum cytokines and synovial microbiota composition in rats after synovial fluid intervention.** (A) Establishment of the knee OA rat model. (B–F) Serum levels of IL-1β, IL-6, TNF-α, MMP-13, and CTX-II (n = 3 per group). (G) Pielou’s evenness index for alpha diversity (n = 6 per group). (H) Beta diversity analysis (n = 6 per group). (I) Phylum-level relative abundance of Firmicutes (n = 6 per group). (J–M) Genus-level comparative abundances of Halomonas, Salinicoccus, Staphylococcaceae_Staphylococcus, and Streptococcus (n = 6 per group). (N) LEfSe-derived histogram of differentially abundant taxa (LDA score > 2; n = 6 per group). C: Control; M: OA model; MA: Antibiotic-treated; MAJ: Antibiotic + synovial fluid intervention. Data are presented as mean ± SD; statistical analysis by one-way ANOVA. *p < 0.05, **p < 0.01, ***p < 0.001.


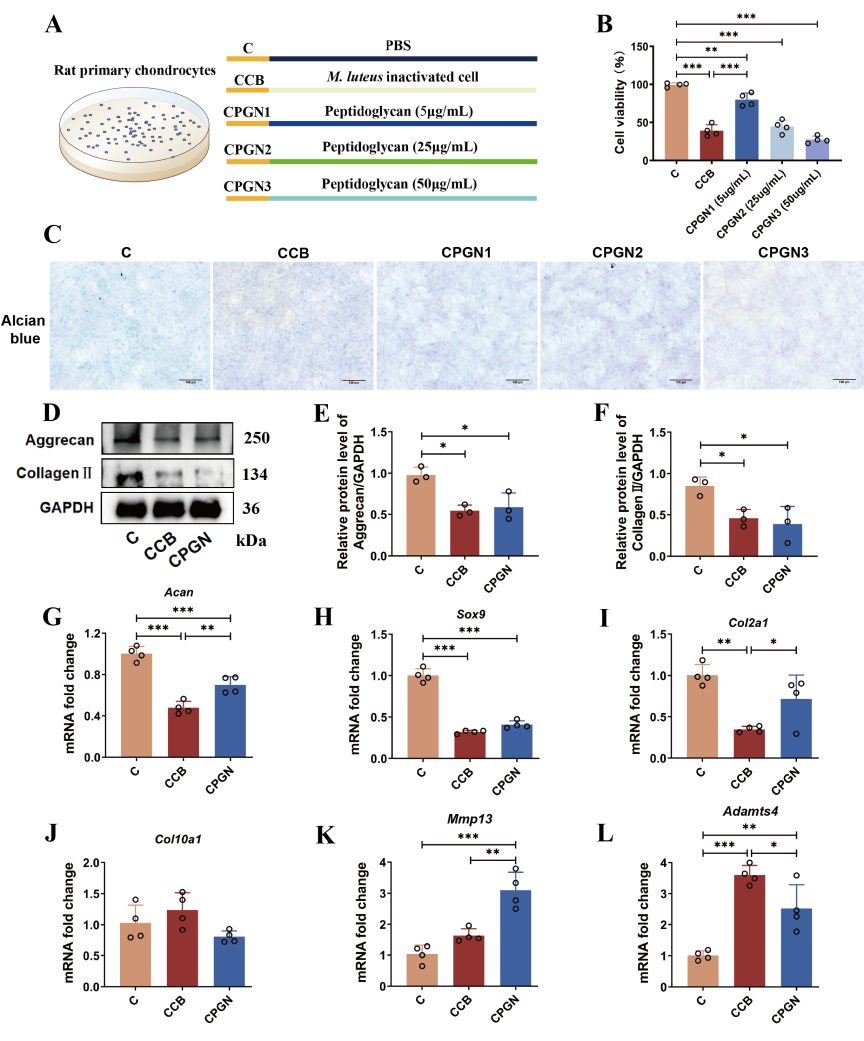


**Figure S3. Peptidoglycan from M. luteus is the key effector aggravating knee OA in rats.** (A) Experimental design for peptidoglycan intervention. (B) Chondrocyte viability after peptidoglycan treatment (n = 4). (C) Alcian blue staining of chondrocytes following peptidoglycan exposure. (D–F) Protein expression of Aggrecan and Collagen II in chondrocytes post-treatment (n = 3). (G–L) mRNA levels of anabolic (Acan, Sox9, Col2a1) and catabolic (Col10a1, Mmp13, Adamts4) genes in peptidoglycan-treated chondrocytes (n = 4). C: Control; CCB: heat-inactivated M. luteus G18 cells; CPGN1–3: peptidoglycan at 5, 25, and 50 µg/mL; CPGN: peptidoglycan at 25 µg/mL. Data are presented as mean ± SD; statistical analysis by one-way ANOVA. *p < 0.05, **p < 0.01, ***p < 0.001.


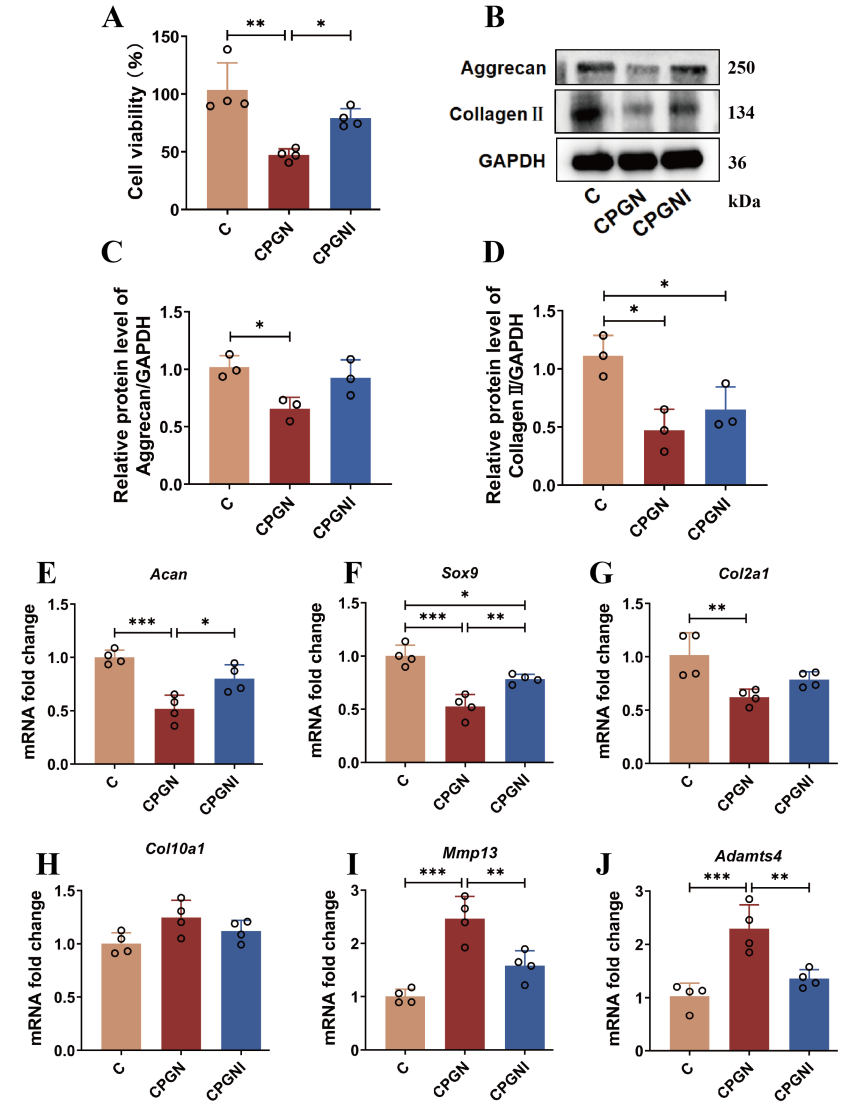


**Figure S4. JNK pathway inhibition mitigates the deleterious effects of peptidoglycan on chondrocytes.** (A) Chondrocyte viability after inhibitor treatment (n = 4). (B–D) Protein expression of Aggrecan and Collagen II in chondrocytes following inhibitor intervention (n = 3). (E–J) mRNA levels of anabolic (Acan, Sox9, Col2a1) and catabolic (Col10a1, Mmp13, Adamts4) genes after inhibitor treatment (n = 4). C: Control; CPGN: peptidoglycan (25 µg/mL) from M. luteus; CPGNI: peptidoglycan + SP600125 (JNK inhibitor). Data are presented as mean ± SD; statistical analysis by one-way ANOVA. *p < 0.05, **p < 0.01, ***p < 0.001.


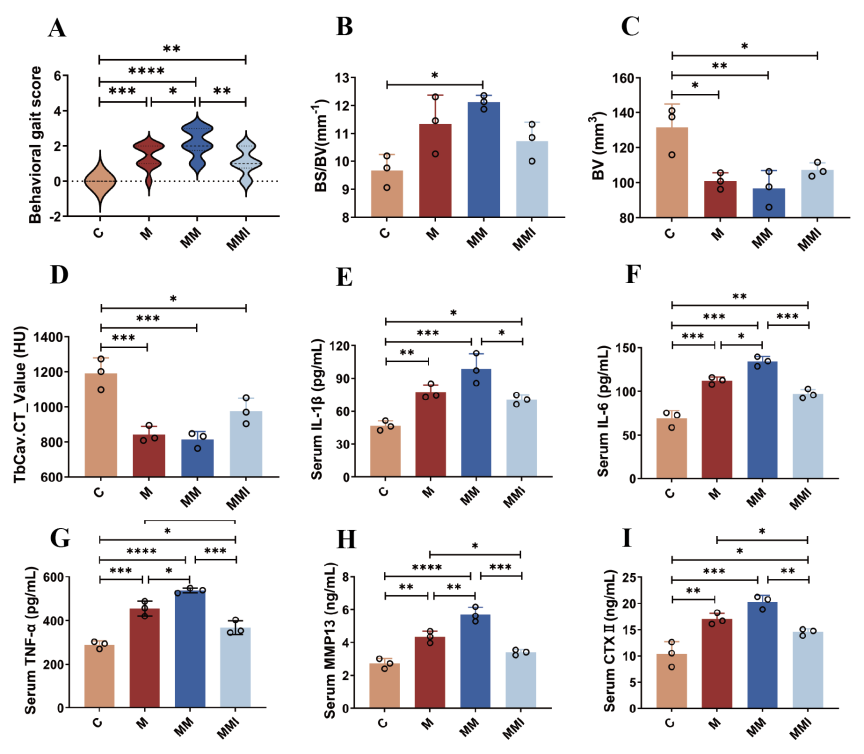


**Figure S5. Effects of M. luteus G18 and JNK inhibition on gait, bone parameters, and serum cytokines in OA rats.** (A) Gait scores (n = 10 rats/group). (B–D) Bone-related parameters in knee joints, including bone surface to bone volume ratio (BS/BV), bone volume (BV), and marrow cavity CT value (TbCav.CT_Value). (E–I) Serum levels of IL-1β, IL-6, TNF-α, MMP-13, and CTX-II. C: Control; M: OA model; MM: M. luteus G18; MMI: M. luteus G18 + SP600125 (JNK inhibitor) (n = 3 per group). Data are presented as mean ± SD; statistical analysis by one-way ANOVA. *p < 0.05, **p < 0.01, ***p < 0.001.

**Table S1. Demographic and clinical characteristics of study participants**

|  | **Severe group (N=20)** | **Mild group (N=20)** | ***p*-value** |
| --- | --- | --- | --- |
| **Age, y, Median (P25, P75)** | 58.50 (54.25, 61.50) | 56.50 (51.00, 59.75) | *p*=0.480 |
| **Sex, N (％)** |  |  | *p*=0.235 |
| Male | 2 (10) | 6 (30) |  |
| Female | 18 (90) | 14 (70) |  |
| **BMI (kg/m^2^), Mean±SD** | 21.75±1.84 | 22.86±2.20 | *p*=0.089 |
| **Physical activity, N (％)** |  |  | *p*=0.803 |
| Often | 3 (15) | 4 (20) |  |
| Sometimes | 3 (15) | 4 (20) |  |
| Seldom or never | 14 (70) | 12 (60) |  |
| **Smoking status, N (％)** |  |  | *p*=0.723 |
| Currently or formerly | 5 (25) | 6 (30) |  |
| Never | 15 (75) | 14 (70) |  |
| **Walking status, N (％)** |  |  | *p*=0.190 |
| Movement disorder | 8 (40) | 4 (20) |  |
| Discomfort during movement | 12 (60) | 14 (70) |  |
| No significant impact | 0 (0) | 2 (10) |  |
| **Duration of symptoms (months), Median (P25, P75)** | 9 (6.00-22.00) | 1.50 (0.52-5.25) | *p*=0.003 |
| **Kellgren-Lawrence grade, N (％)** |  |  | *p*<0.001 |
| Grade Ⅰ | 0 (0) | 12 (60) |  |
| Grade Ⅱ | 0 (0) | 8 (40) |  |
| Grade Ⅲ | 17 (85) | 0 (0) |  |
| Grade Ⅳ | 3 (15) | 0 (0) |  |
| **More symptomatic knee, N (％)** |  |  |  |
| Left knee | 8 (40) | 8 (40) |  |
| Right knee | 12 (60) | 12 (60) |  |

Data are presented as n (%), mean ± SD, or median (IQR). All comparisons were considered statistically significant at two-tailed p < 0.05. Group differences were assessed using independent samples t-test for normally distributed variables, Mann–Whitney U test for non-normally distributed variables, and Chi-square (χ²) test for non-ordinal categorical variables.

**Table S2. Bacterial culture of synovial fluid**

| **Bacterial culture of synovial fluid** | **Severe group (N=20)** | **Mild group (N=20)** | ***p*-value** |
| --- | --- | --- | --- |
| Number of positive samples**, N (％)** | 22 | | *p*<0.001 |
| Positive | 18 (90) | 4 (20) |  |
| Negative | 2 (10) | 16 (80) |  |
| Number of isolates | 125 | 20 |  |
| Species number | 57 | |  |

**Table S3. Omics analysis of synovial fluid cultures from OA patients**

| **Genus** | **Species** | **Number** |
| --- | --- | --- |
| *Acinetobacter* | *Acinetobacter johnsonii* | 2 |
|  | *Acinetobacter pseudolwoffii* | 1 |
| *Agrococcus* | *Agrococcus jejuensis* | 1 |
| *Bacillus* | *Bacillus cereus* | 1 |
|  | *Bacillus pacificus* | 7 |
|  | *Bacillus subtilis subsp. subtilis str.* | 20 |
|  | *Bacillus toyonensis* | 2 |
|  | *Bacillus wiedmannii* | 1 |
|  | *Metabacillus indicus* | 1 |
| *Brachybacterium* | *Brachybacterium massiliense* | 2 |
|  | *Brachybacterium saurashtrense* | 1 |
| *Brevundimonas* | *Brevundimonas huaxiensis* | 1 |
| *Citricoccus* | *Citricoccus muralis* | 3 |
| *Corynebacterium* | *Corynebacterium glutamicum SCgG2* | 2 |
|  | *Corynebacterium liangguodongii* | 1 |
|  | *Corynebacterium singulare* | 1 |
|  | *Corynebacterium stationis* | 1 |
| *Curtobacterium* | *Curtobacterium citreum* | 1 |
| *Cytobacillus* | *Cytobacillus ciccensis* | 1 |
| *Empedobacter* | *Empedobacter falsenii* | 1 |
| *Escherichia* | *Escherichia coli O157* | 1 |
| *Facklamia* | *Facklamia hominis* | 1 |
|  | *Facklamia tabacinasalis* | 1 |
| *Jeotgalicoccus* | *Jeotgalicoccus meleagridis* | 1 |
| *Kocuria* | *Kocuria flava* | 2 |
| *Lysinibacillus* | *Lysinibacillus capsici* | 2 |
|  | *Lysinibacillus contaminans* | 1 |
| *Mammaliicoccus* | *Mammaliicoccus lentus* | 1 |
|  | *Mammaliicoccus vitulinus* | 1 |
| *Microbacterium* | *Microbacterium chocolatum* | 1 |
| *Micrococcus* | *Micrococcus aloeverae* | 2 |

| **Genus** | **Species** | **Number** |
| --- | --- | --- |
| *Micrococcus* | *Micrococcus endophyticus* | 7 |
|  | *Micrococcus luteus* | 26 |
|  | *Micrococcus yunnanensis* | 3 |
| *Moraxella* | *Moraxella osloensis* | 5 |
| *Nocardiopsis* | *Nocardiopsis algeriensis* | 1 |
| *Ornithinimicrobium* | *Ornithinimicrobium pratense* | 4 |
| *Paenibacillus* | *Paenibacillus aceti* | 1 |
|  | *Paenibacillus odorifer* | 1 |
| *Piscinibacter* | *Piscinibacter defluvii* | 1 |
| *Priestia* | *Priestia aryabhattai* | 1 |
| *Pseudomonas* | *Pseudomonas rhizoryzae* | 3 |
|  | *Pseudomonas vanderleydeniana* | 1 |
| *Rhodococcus* | *Rhodococcus qingshengii* | 6 |
| *Roseomonas* | *Roseomonas mucosa* | 1 |
| *Ruoffia* | *Ruoffia halotolerans* | 1 |
| *Staphylococcus* | *Staphylococcus cohnii* | 1 |
|  | *Staphylococcus epidermidis* | 2 |
|  | *Staphylococcus equorum* | 2 |
|  | *Staphylococcus hominis* | 2 |
|  | *Staphylococcus microti* | 1 |
|  | *Staphylococcus nepalensis* | 3 |
|  | *Staphylococcus pseudoxylosus* | 3 |
|  | *Staphylococcus warneri* | 1 |
| *Streptococcus* | *Streptococcus pluranimalium* | 1 |
| *Suicoccus* | *Suicoccus acidiformans* | 1 |
| *Tessaracoccus* | *Tessaracoccus flavescens* | 1 |

**Table S4. Gait scoring criteria for OA in rats**

| **Score** | **Description** | **Specific Manifestations** |
| --- | --- | --- |
| 0 | Normal gait | Both hindlimbs exhibit uniform ink staining. |
| 1 | Mild lameness | Each step shows toe and partial heel staining without limb dragging or lifting. If the left heel staining is minimal (indicating toe-walking), right toe staining is reduced by ~25% compared to the left. |
| 2 | Moderate lameness | Only toe staining is observed in all steps, without dragging or lifting. If left heel staining remains minimal, right toe staining is reduced by ~50% relative to the left. |
| 3 | Severe lameness | Limb dragging or lifting is evident, with black drag marks on the dorsum or at least one step showing minimal toe contact. In some cases, the rat attempts a single step using the right foot with ~75% reduction in staining compared to the left. |
| 4 | Extreme lameness | The affected limb is not used during walking, with either complete absence of staining or only faint drag marks without toe contact, indicating ~100% reduction in right limb staining. |

Higher scores indicate more severe joint damage.

**Table S5. List of antibodies used in this study**

| **Antibodies** | **Source** | **Cat. No.** |
| --- | --- | --- |
| Aggrecan | proteintech | 13880-1-AP |
| Collagen II | proteintech | 28459-1-AP |
| Cleaved-Caspase-3 | CST | 9661S |
| Caspase-3 | CST | 9662S |
| Bax | proteintech | 50599-2-Ig |
| Bcl-2 | proteintech | 68103-1-lg |
| TLR2 | ABCAM | ab213676 |
| p-JNK | CST | 9255S |
| JNK | CST | 9252S |
| p-c-fos | CST | 5348T |
| c-fos | proteintech | 66590-1-Ig |
| GAPDH | proteintech | 60004-1-Ig |

**Table S6. Primer Sequences**

|  | **Forward Primer** | **Reverse Primer** |
| --- | --- | --- |
| *Acan* | AATGGGAGCCAGCCTACAC | TTGAGAGGCAGAGGGACTTT |
| *Sox9* | TGAAGAAGGAGAGCGAGGAA | CATAGCCCTTCAGCACCTG |
| *Col2a1* | CCAGGTCCTGCTGGAAAA | CCTCTTTCTCCGGCCTTT |
| *MMP13* | TGGACAAGCAGCTCCAAAG | GTCCAGACCGAGGGAGTG |
| *ADAMTS-4* | GTACCTACCTGACTGGCACCATC | TGCTGCCATCTTGTCATCTGC |
| *Col10a1* | CACAGCCATTTCGAGCTTTT | TCTAAGTTGCCCCAGGTACG |
| *TLR2* | TCACTGTTCTCCAATCTCACAA | CAGCCCAGCAAAATCTATTCTC |
| *TLR4* | CCGCTCTGGCATCATCTTCA | CTCCCACTCGAGGTAGGTGT |
| *NOD2* | GTTCTGATGGGGGTCAGTGG | GACGGCCTTAGAGGGTAAGC |
| *ERK* | TAGGCATCCGAGACATCCTC | TCGCAGGTGGTGTTGATAAG |
| *AMPK* | TGTCTGCCGTGGACTACTGT | GCTGCATAATTTGGCGATC |
| *AKT* | CTTTATTGGCTACAAGGAACG | AGTCTGAATGGCGGTGGT |
| *JNK* | GATTTGGAGGAGCGAACTAAG | TGCTGTCTGTATCCGAGGC |
| *NF-κB* | AGAGAAGCACAGATACCACTAAGA | GTTCAGCCTCATAGAAGCCATC |
| *GAPDH* | CTGGAGAAACCTGCCAAGTATG | GGTGGAAGAATGGGAGTTGCT |
